# Nanogold Foundry Involving High‐Shear‐Mediated Photocontact Electrification in Water

**DOI:** 10.1002/smsc.202300312

**Published:** 2024-05-09

**Authors:** Badriah M. Alotaibi, Zoe Gardner, Kasturi Vimalanathan, Xianjue Chen, Thaar M. D. Alharbi, Colin L. Raston

**Affiliations:** ^1^ Flinders Institute for Nanoscale Science and Technology College of Science and Engineering Flinders University Adelaide SA 5042 Australia; ^2^ School of Environmental and Life Sciences The University of Newcastle Callaghan NSW 2308 Australia; ^3^ Physics Department, Faculty of Science Taibah University Almadinah Almunawarrah 42353 Saudi Arabia

**Keywords:** mechanoenergies, nanogolds, photocontact electrification, topological fluidic flows, vortex fluidic devices

## Abstract

Controlling the size and morphology of gold nanoparticles occurs in the absence of added reducing agents or other excipients such as surfactants, on UV irradiation (*λ* 254 nm) of aqueous auric acid (H[AuCl_4_]) in a thin film of liquid in a vortex fluidic device (VFD) within a rapidly rotating tilted quartz tube. This involves contact electrification (CE), which occurs at the solid−liquid interface with the oxidation of water photoinduced, forming the hydroxyl radical, OH^•^. In air, the redox couple is reduction of ^3^O_2_ to the superoxide radical anion, O_2_^−•^, which then reduces Au^3+^ to elemental gold, as does other reactive oxygen species present, competing with CE reduction of Au^3+^. The resulting nanogold structures effectively mold the different high‐shear topological fluid flows in the VFD, being isolated as ultrathin 2D sheets, prisms, hierarchical structures comprising nanoparticles embedded within these sheets, and rosette and tubular structures, depending on the VFD processing parameters and the concentration of auric acid. Processing under a nitrogen atmosphere while UV irradiated affords mainly 2D gold through the above reduction of Au^3+^. The findings establish a paradigm for VFD processing in water involving photo‐CE, generating hydrogen peroxide and hydrogen gas with the surfaces of the gold nanoparticles pristine.

## Graphical abstract

The size and morphology of pristine gold nanoparticles is controlled by UV irradiation of aqueous auric acid in a vortex fluidic device housing a tilted rapidly rotating tube, as a photocontact electrification process at the solid−liquid interface, involving oxidation of water coupled to reduction of Au^3+^ competing with O_2_ forming O_2_^−•^ which then reduces Au^3+^.

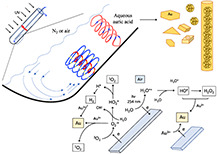

## 1 Introduction

Controlling the size and shape of gold nanoparticles is critical for a diversity of applications, including in drug delivery, catalysis, sensing, and electronics, due to their unique physical, chemical and optical properties relative to bulk metal.^[^ ^1^, ^2^, ^3^ ^]^ Gold nanoparticles can be synthesized using physical or biological methods,^[^ ^4^, ^5^, ^6^ ^]^ with chemical methods being the most widely used because of the ease of functionalization and the ability to control their size and morphology. Typically, chemical methods use aqueous solution of gold(III), such as tetrachloroaurate, and various reducing agents, for example, sodium citrate, sodium tetrahydroborate, and thiol compounds, which may also act as stabilizers for the resultant nanoparticles.^[^ ^3^ ^]^ However, making thin 2D gold material using wet chemical methods is challenging because of the highly isotropic lattice symmetries of the metal disfavoring anisotropic crystal growth. As a result, regulating the crystal growth requires reducing surface energy using capping agents such as poly(vinyl pyrrolidone) (PVP),^[^ ^7^ ^]^ which prevent specific facet growth of seed crystals, favoring directed growth of nanosheets. Other effective capping agents for such growth include graphene oxide,^[^ ^8^ ^]^ methyl orange (MO),^[^ ^9^ ^]^ and a phosphinated calix[8]arene.^[^ ^10^ ^]^ The synthesis of nanoparticles of gold can also involve the use of external fields such as laser light sources,^[^ ^11^ ^]^ microwaves,^[^ ^12^ ^]^ and sonication,^[^ ^13^ ^]^ with recent studies on plasma‐induced electrification technology.^[^ ^14^ ^]^ There is also the use high temperatures and pressures and long processing times.^[^ ^15^ ^]^ The scalability of any process is important^[^ ^15^ ^]^ which can benefit from continuous flow reactors, with nanorods, for example, formed using a sequential rotating tube processor (RTP) and narrow channel processing systems.^[^ ^16^ ^]^ The RTP has a horizontally aligned rotating tube with liquids delivered at one end of the tube, which then whirl along the tube, exiting at the other end. It was the forerunner for development of the vortex fluidic device (VFD).

**Figure 1:**
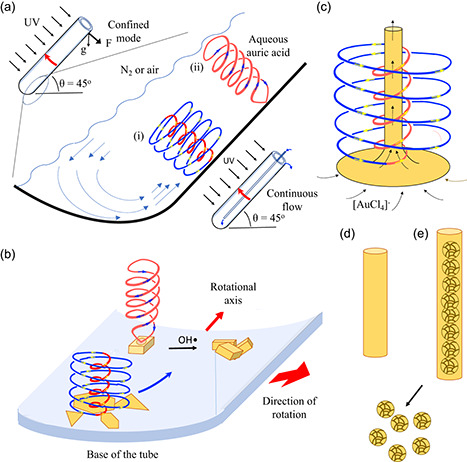
a) Schematic of the VFD showing its confined and continuous flow modes of operation, forming i) typhoon‐like ST topological fluid flow from the Coriolis force from the hemispherical base of the rapidly rotating tube and ii) DH flow from the induced Faraday wave eddies coupled with the Coriolis force from the curve surface of the tube.^[^ ^17^, ^18^ ^]^ b) Confined‐mode UV (*λ* = 254 nm)‐induced formation of 2D gold sheets on the surface of the quartz at the base i) at 5 k rpm, and prisms of gold with divots formed at the base ii) at 7 k rpm, with the holes absent on further processing. c) Formation of continuous hollow tubes of gold from the surface of the VFD tube by i) affording d) hollow tubes of gold or e) tubes of gold confining gold rosettes, with breaching of the thin gold tubes affording free‐standing rosettes. All processing outcomes are at *θ* = 45° and depend on the concentration of auric acid, ω, mode of operation of the VFD, and flow rate under continuous flow.

The VFD has a rapidly rotating tube open at one end with liquids delivered to the tube through jet feeds, to the base (closed end) of the tube or indeed at any position along the tube, with the tube tilted relative to the horizontal position such that liquids exit at the top of the tube, as shown in **Figure** **1**.^[^ ^17^, ^18^ ^]^ Optimal processing for the many and varied applications of this thin film microfluidic platform is usually with a tilt angle, *θ*, of 45°, whether the device is operated under continuous flow conditions, or the confined mode of operation, where a finite volume of liquid is processed for a specific time. The other primary operating parameter of the VFD is the rotational speed, *ω*, which for the standard 20 mm‐outside diameter (OD) tube (≈17.5 internal diameter [ID] and 18.5 cm in length) is between 1500 and 9000 rpm.^[^ ^17^, ^18^ ^]^ Depending on the nature of the liquid, either as a homogeneous phase or a mixture of immiscible solvents where mixing is at the molecular level, the induced mechanoenergy in the liquid is in the form of high‐shear topological fluid flows down to submicrometer dimensions. The film is uniform in thickness along the upper side of the tube when the tube is tilted at 45°, which is consistent with the presence of Faraday waves. The high‐shear stress regimes are in the form of 1) typhoon‐like spinning top (ST) flows arising from the Coriolis force from the hemispherical base of the tube, 2) double‐helical (DH) flows arising from the side wall Coriolis force twisting of Faraday wave eddies, and 3) a resonant spicular or spherical flow.^[^ ^18^ ^]^ A detailed understanding of the induced high shear mechanical energy in liquids in the VFD, arising from the effect of gravity interplaying with centrifugal force, is important in controlling any process. Applications of the VFD include immunoblot assay analysis,^[^ ^19^ ^]^ chemical synthesis,^[^ ^20^ ^]^ accelerating enzymatic reactions,^[^ ^21^ ^]^ protein folding,^[^ ^22^ ^]^ materials processing,^[^ ^23^ ^]^ food processing,^[^ ^24^ ^]^ and self‐assembly processes.^[^ ^25^ ^]^ Processing in the VFD can involve applying fields such as light sources for which the VFD is ideally suited in having a thin film of liquid down to a thickness of ≈200 μm.^[^ ^26^ ^]^ The VFD is also suited for real‐time monitoring, including using UV−vis and fluorescence spectroscopy, neutron imaging, and small‐angle neutron scattering.^[^ ^27^ ^]^

We hypothesized that the high shear regimes in the VFD can lead to contact electrification (CE), including when applying external fields, for the in situ reduction of gold. CE is a quantum mechanical effect occurring at liquid−gas (L−G), liquid−liquid (L−L), and solid–liquid (S−L) interfaces with electron transfer being the dominant mechanism,^[^ ^28^ ^]^ although there is no universal mechanism of understanding the effect. All liquids form an electrical double layer at the interface of liquids and solids,^[^ ^28^ ^]^ which is assumed to be the primary surface for CE, with recent studies supporting the presence of CE for L−L and L−G interfaces.^[^ ^28^, ^29^ ^]^ Water is not necessary for CE, although H^+^ and OH^−^ ions from interfacial water may transfer charges between surfaces.^[^ ^28^ ^]^ CE is important for accelerating many interfacial chemical, biochemical, and catalytic processes.^[^ ^28^ ^]^ It occurs when the interatomic separation between two atoms/molecules/materials is forced to a distance less than the normal bonding length.^[^ ^28^, ^30^ ^]^ In this context the mechanoenergy delivered in the VFD within the topological fluid flows is likely to result in CE, noting that localized temperature and pressures generated in the VFD can be very high. The VFD lends itself to enhancing CE by varying the surface area of the tube which is possible, for example, by coating the tube with xerogel silica and polymers,^[^ ^31^ ^]^ noting that there is high mass transfer into a porous surface matrix on the surface of the tube, driven by the typhoon‐like ST topological fluid flow. Synthesis based on CE involves sonication^[^ ^28^, ^29^ ^]^ where collapse of the induced cavitation bubbles occurs, as random events in time and place.^[^ ^28^ ^]^ Any CE associated with high‐shear topological fluid flows in the VFD is likely to be more controlled, and this could make the VFD a more attractive platform for harnessing the phenomenon of CE. The mechanism of CE on L−G interfaces is poorly understood, with studies focused on levitating a liquid droplet with ultrasound generating charges on the surface of the droplets.^[^ ^32^ ^]^

**Figure 2:**
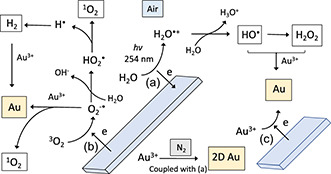
Proposed mechanism for the formation of gold particles involving a) photo‐CE‐induced oxidation of water to OH^•^ and hydrogen peroxide coupled with b) reduction of in situ‐generated O_2_^−•^ and/or c) Au^3+^, with the latter resulting in formation of mainly 2D gold in high yield under nitrogen atmosphere; reactive oxygen species (ROS) detected, along with H_2_, are highlighted in color.

Herein we establish electron transfer in water during CE for driving chemical reactions without the need for adding reagents which would otherwise be required. Processing of an aqueous solution of auric acid (H[AuCl_4_]) in the VFD under an atmosphere of air while the rotating quartz tube is irradiated at *λ* = 254 nm, as shown in Figure 1, affords different sizes and shapes of nanoparticles of gold which are essentially a mold of the topological fluid flows in the liquid when the concentration of auric acid is varied. The reduction of auric acid involves photo‐CE,^[^ ^33^ ^]^ with water oxidized to H_2_O^+•^ enroute to the hydroxyl radial, OH^•^, and hydrogen peroxide, coupled with 1) reduction of oxygen to the superoxide radical anion, O_2_^•−^, and 2) reduction of Au^3+^ to elemental gold, as established by carrying out processing under nitrogen atmosphere which shuts down the formation of O_2_^•−^, as shown in **Figure** **2**. The discovery of CE in the VFD in water advances the syhtesis of gold nanoparticles with high green chemistry metrics, as well as being able to generate hydrogen rather than a mixture of hydrogen and oxygen from water which has implications in the energy sector.^[^ ^34^ ^]^ This is without the need for a catalyst, unlike in photocatalytic generation of hydrogen.^[^ ^35^ ^]^ Also noteworthy is 1) the ability to generate hydrogen peroxide in situ via CE without requiring catalysts for green chemical oxidation processes, offering tantalizing possibilities in nanomaterials synthesis and in organic chemistry.^[^ ^36^ ^]^

## 2 Results and Discussions

### 2.1 Formation of Gold Nanomaterial in Air

**Figure 3:**
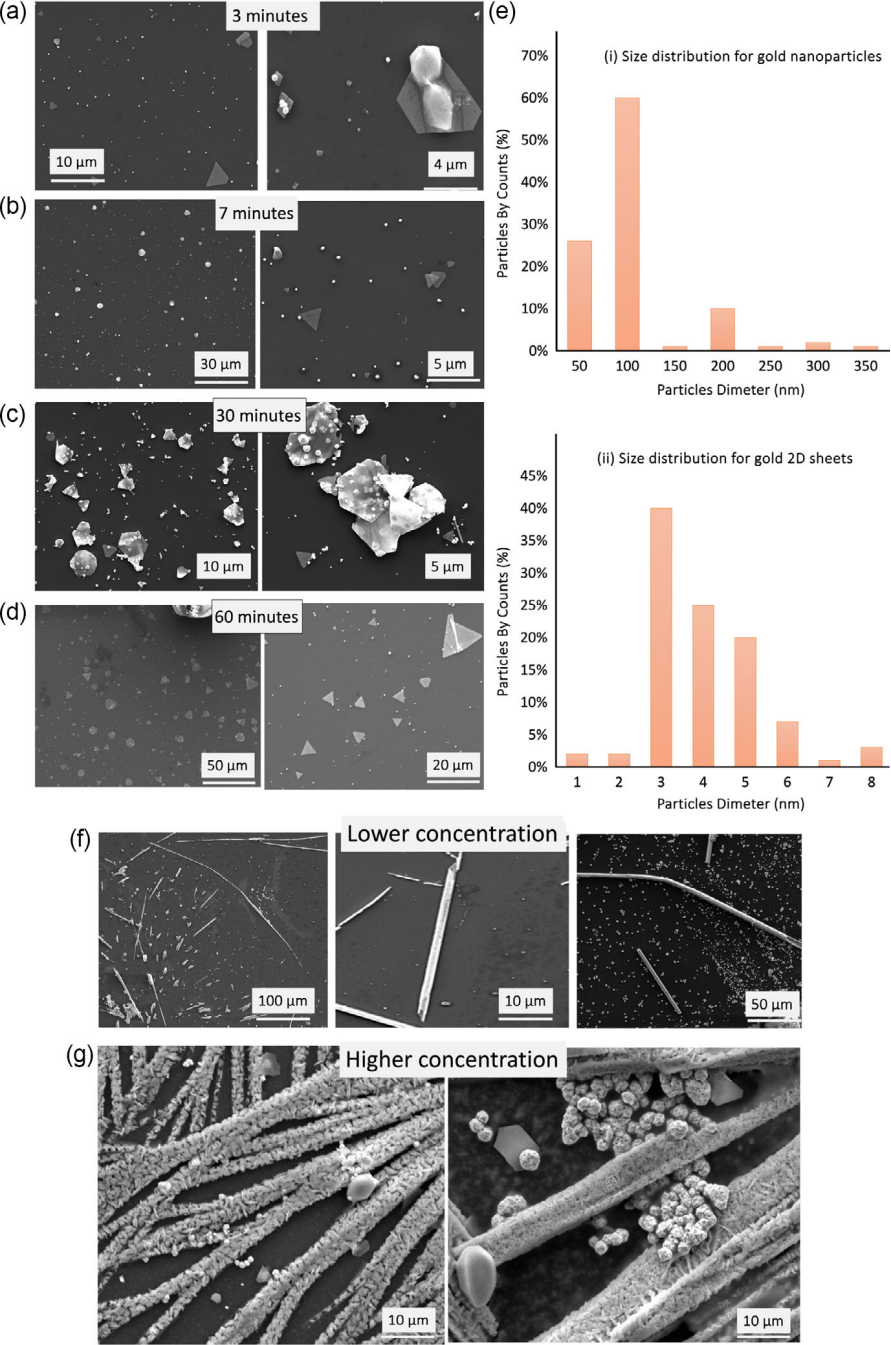
SEM images of gold nanoparticles formed after VFD processing under atmosphere of air for a) 3, b) 7, c) 30, and d) 60 min processing, forming ultrathin 2D triangular and hexagonal sheets, concentration of auric acid 3.7 mm, e) particle size distribution for i) gold nanoparticles and ii) 2D triangular sheets, f) SEM images of rods and hollow tube‐like structures of gold formed at a lower concentration, 3.0 mm, g) SEM images for higher auric acid concertation, 5.0 mm, forming composites of ultrathin sheets of gold encapsulating rosette‐shaped nanostructures. VFD processing was as follows: *ω* = 5 k rpm, *θ* = 45°, 60 min, volume = 1 mL, confined mode, UV irradiation, *λ* = 254 nm.

VFD processing was initially in the confined mode, under an atmosphere of air in a standard 20 mm OD (17.5 mm ID) quartz tube tilted at 45° with the rotational speed, *ω*, set at 5 k rpm as a representative speed for water where the shear stress is dominated by both typhoon‐like ST flow and DH topological fluid flows, as shown in Figure 1a,^[^ ^18^ ^]^ with the concentration of auric acid at 3.7 mm. This resulted in only small amounts of gold nanomaterial, mainly as thin sheets, as shown in Figure S2b, Supporting Information. Irradiating the VFD tube while rotating at 5 k rpm with UV (*λ* = 254 nm) resulted in the formation of gold nanomaterial, in a way dramatically depending on the processing time, as shown in **Figure** **3** and S3, Supporting Information. After 3 and 7 min, spheroidal particles, ≈0.2 μm diameter, and 2D triangular sheets, ≈1.6 μm edges, were present, as shown in Figure 3a,b. The spheroidal particles were also present after 30 min of processing, as shown in Figure 3c, but now with larger 2D triangular sheets, and hexagonal sheets, ≈5 μm along the edges, with these sheets also having small particles, ≈0.2μm in diameter, attached to them. After 60 min, these particles underwent dissolution and regrowth, forming much larger 2D triangular and hexagonal sheets, with their sizes ranging in cross section from ≈5 μm, as shown in Figure 3d. This dissolution and regrowth is consistent with the formation of OH^•^ formed by CE, as shown in Figure 2a, which is known to dissolve gold^[^ ^37^ ^]^ and is further discussed below. The preferential growth into 2D sheets in the absence of surfactants presumably arises from the high shear, resulting in dissolution and regrowth occurring on the surface of the tube. Size distributions of gold nanoparticles formed after 60 min processing are shown in Figure 3e, with the diameter of spherical particles centered at 100 nm and triangles with edges between ≈3 and 5 μm. Solutions of gold nanoparticles prepared in the VFD exhibit stability over a month at ambient conditions with zeta potentials ≈−11 mV along with a small peak at −40 mV, as shown in Figure S13c, Supporting Information. The initial pH of an auric acid solution before VFD processing was 1.8, dropping to 1.5 after 60 min of VFD processing, which is consistent with CE affording [H_3_O]^+^ (and OH^•−^) and direct CE reduction of Au^3+^, as shown in Figure 1 and 2.

VFD processing in the absence of UV irradiation, for the otherwise optimized conditions for generating gold nanomaterials in the confined mode, gave low yields, 10%, of gold nanomaterials, as shown in Figure S2b, Supporting Information, as judged using scanning electron microscopy (SEM). As another control, UV irradiation of solutions of auric acid without VFD processing also gave only small amounts of gold nanoparticles, 10%, Figure S2c, Supporting Information. In contrast, for the same concentration of auric acid with the VFD operating in the confined mode in air under UV irradiation, changing *ω* resulted in distinctly different outcomes after 60 min processing. For *ω* = 7 k rpm, the initial product was gold prisms with divots, and with further processing, these prisms became devoid of the divots, Figure S4, Supporting Information. The initial divots presumably arise from the formation of gold around the high shear areas where DH flow strikes the surface of the glass tube, noting that prism with divots also occurs in preparing metal organic frameworks in the VFD.^[^ ^18^ ^]^ For *ω* = 4 k rpm, under the same conditions as for the other two speeds, rods along with ultrathin sheets of gold formed, Figure S5, Supporting Information. The results thus far establish that *ω* = 5 k rpm is effective for forming the 2D triangular and hexagonal sheets, after 60 min of processing, and subsequently this rotational speed was fixed with the concentration of auric acid varied, also while operating the VFD in the confined mode for 60 min while irradiated with UV, with *θ* at 45°. This resulted in the formation of a variety of different types of nanoparticles, Figure 3a–g, as established using SEM images, as follows: 1) 2D triangular and hexagonal sheets, Figure 3a–d, 2) hollow tube‐like structures, Figure 3f, and 3) composites of ultrathin sheets of gold incorporating small nanoparticles, Figure 3g.

Ultrathin sheets of gold formed at *ω* = 5 k rpm for both 3.7 and 4.0 mm concentrations of auric acid after 60 min processing time, as shown in Figure 3d and S6e, Supporting Information. For 3.0 mm concentration, hollow tubes formed, Figure 3f, which are unstable, Figure S6c, Supporting Information, undergoing Oswald ripening in water and on a silicon wafer, as shown in Figure S6c, Supporting Information, in the absence of residual auric acid, forming sheets organized into tubular structures. The formation of the above hollow tubes provides mechanistic insight for gold dissolving and redepositing, forming gold prisms, Figure S6c, Supporting Information, which is further discussed later. Higher concentrations of auric acid, 5.0, 7.0, and 13.0 mm, resulted in the formation of ultrathin side‐walled tubes encapsulating rosette‐shaped nanoparticles, Figure 3g and S6e,f, Supporting Information. These tubes are connected into extended structures for which their length is much longer than the thickness of the film of liquid, ≈250 μm at *ω* = 5 k rpm.^[^ ^18^ ^]^ This is ascribed as arising from the unique ST topological fluid flow generated from the hemispherical base of the tube constantly weaving the gold into tube‐like structures, as “peas‐in‐a‐pod.” For concentrations of auric acid ≥7.0 mm, with *ω* 5 k rpm and the VFD tube irradiated (*λ* = 254 nm), ultrathin pipes surrounding the irregular‐shaped gold particles are formed, Figure S6e,f, Supporting Information. The formation of the ultrathin pipes is ascribed to deposition of gold on the inner wall helical flow up the center of the ST. The rosettes formed inside these gold tubes (pipes) are likely to be a cast of the fluid flow in the center of the ST. At high concentration of auric acid, the rosettes in thin pipe structures are constantly produced in the “spinning wheel” inside the ST, and these structures can be exceptionally long, >1 mm, much larger than the thickness of the film in the VFD.^[^ ^18^ ^]^ Determining the height of the ST relative to the thickness of the film remains an open research question. As for determining the outer diameter of the ST, especially where the liquid is spiraling down onto the surface of the quartz tube, advances have been made, through melting of bismuth metal in an organic solvent in forming craters, resulting from the high shear and localized heating on the surface of the tube, as well as drilling into preformed polymer films on the inner surface of the VFD tube.^[^ ^6^, ^14^ ^]^

Replacing the hemispherical‐based tube with a flat base, with the VFD operating under the above‐optimal confined mode conditions at *ω* = 5 k rpm, resulted in rods rather than ultrathin sheets of gold, as shown in Figure S7a, Supporting Information. The effect of change in pH of the auric acid solution pre‐VFD processing for the hemispherical‐based tube was also explored using the confined mode of operation of the VFD while keeping all the other optimized confined mode processing parameters constant. Adjusting the pH to 9.0 by adding aqueous 0.1 M sodium hydroxide resulted in the formation of ≈5 μ*m*‐diameter gold prisms in the VFD while UV irradiated, as shown in Figure S7b, Supporting Information, and the same occurred for *θ* = 0°, Figure S7c, Supporting Information, instead of 45°. For comparison, sonication of gold solutions rather than VFD processing resulted in some nanoparticles with and without UV irradiation, but they are irregular in shape. For VFD processing in air, the optimized parameters for generating gold nanosheet use a hemispherical‐based tube, *θ* = 45°, *ω* = 5 k rpm, VFD tube irradiated (*λ* = 254 nm), concentration of auric acid from 3.7 to 4 mm, and pH = 2.

### 2.2 Characterizations of 2D Gold Sheets

**Figure 4:**
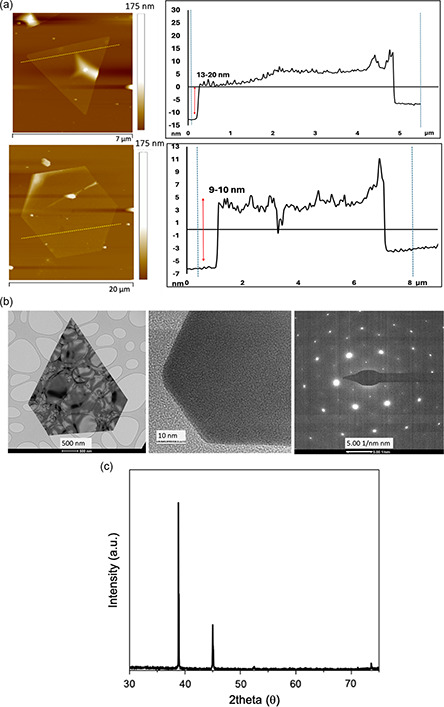
a) AFM images of triangular and hexagonal ultrathin gold sheets with a height (thickness) of 5−10 nm. b) TEM images for triangular gold sheets with the SAED pattern. c) XRD pattern for gold nanoparticles. The VFD processing was as follows: *ω* = 5 k rpm, *θ* = 45°, 60 min, volume = 1.0 mL, concentration = 3.7 mm, irradiation at *λ* = 254 nm.

**Figure 5:**
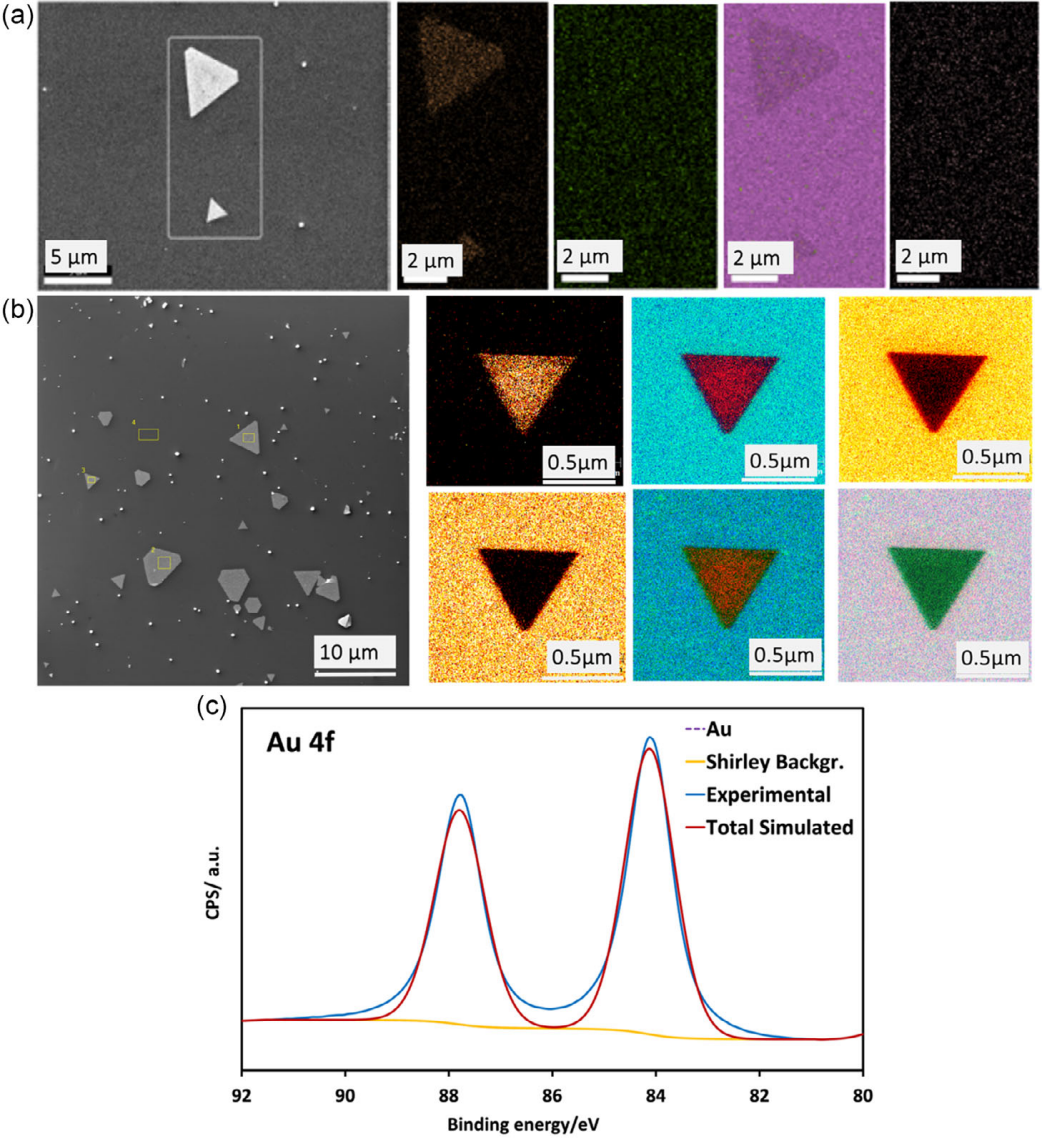
a) SEM/EDX images of the corresponding Au sheet with elemental mapping images for Au, O, Cl, and Si. b) SEM/AES images of the corresponding Au sheet with elemental mapping images (iii‐viii) for O, C, and Si. (b) Color code for AES: red (gold), blue (oxygen), orange (carbon), and green (silicon). c) XPS spectra gold nanoparticles. The VFD processing was as follows: *ω* = 5 k rpm, *θ* = 45°, 60 min, 1.0 mL, 3.7 mm auric acid solution, irradiation at *λ* = 254 nm.

A striking feature of the 2D gold sheets formed at 3.7 mm concentration of auric acid, with ω 5 k rpm and under UV irradiation, is their uniformity of shape, having well‐defined triangular and hexagonal structures, and they can fold, Figure 3d, highlighting their fragility as ultrathin sheets. They range in thickness from 5 to 10 nm (average 8 nm) as established using atomic force microscopy (AFM) imaging, as shown in **Figure** **4**a and S12, Supporting Information. Gold sheets with similar thickness can be prepared as a templating process using phosphonated calix[8]arene over 10 h^[^ ^10^ ^]^ in contrast to minutes in the present study in the absence of any templating agent. Transmission electron microscopy (TEM) established the size of the triangular and hexagonal sheets along their edges, ≤500 nm, as shown in Figure 4b, which is consistent with SEM results, as shown in Figure 3e(ii). Selected‐area electron diffraction (SAED) pattern of a large sheet shows spots with hexagonal symmetry Figure 4b, consistent with face centred cubic (fcc) gold sheets orientated on the <111> face with an interlayer spacing of 0.25 nm, corresponding to the 1/3(422) facet of Au crystals,^[^ ^10^ ^]^ and this is also consistent with X‐ray diffraction (XRD) patterns, as shown in Figure 4c. In general, fcc is the preferred structure for single‐crystal nanoparticles whereas hexagonal‐close‐packed (hcp) is prevalent for polycrystalline nanoparticles of gold.^[^ ^38^ ^]^ In addition, the nucleation and growth of gold particles, for example, nanorods, can involve a seeded growth mechanism in forming hcp gold.^[^ ^38^ ^]^ The composition of the 2D sheets was probed using energy‐dispersive X‐ray spectroscopy (EDX), Auger, and X‐ray photoelectron spectroscopy (XPS), as shown in **Figure** **5**a–c and S13a,b, Supporting Information. Prominent SEM/EDX peaks on the time‐dependent samples were assigned to Au, which is consistent with the sheets being mainly composed of gold, as shown in Figure 5a. Auger electron spectra (AES) of the 2D sheets, Figure S13b, Supporting Information, with elemental mapping images for O, C, and Si are shown in Figure 5b, showing that the material is mainly comprised of gold. XPS spectra is shown in Figure 5c, revealing two peaks for 4*f* gold with the binding energy for elemental old, at 84.22 and 87 eV, which is in good agreement with the binding energy for gold in the literature.^[^ ^39^, ^40^, ^41^ ^]^

### 2.3 Continuous Flow Processing

For translating the confined mode of processing into continuous flow in targeting the above triangular and hexagonal 2D sheets, a flow rate of 0.5 mL min^−1^ for a 3.7 mm solution was initially used, being typically the optimized flow rate for several other applications of the VFD.^[^ ^14^ ^]^ This resulted in the formation of hollow tubes along with other structures, Figure S8a, Supporting Information, with the flow rate corresponding to a residence time of several minutes.^[^ ^27^ ^]^ This is much shorter than 60 min optimal processing time for the confined mode of operation of the VFD, for generating triangular and hexagonal 2D sheets for a 3.7 mm auric acid solution. Scaling up the process for generating uniform triangular and hexagonal sheets resulted in ≈60% yield for lower flow rates of 0.2 and 0.3 mL min^−1^, Figure S8b, Supporting Information, which dramatically extends the residence times.

#### 2.3.1 Processing Under Nitrogen

**Figure 6:**
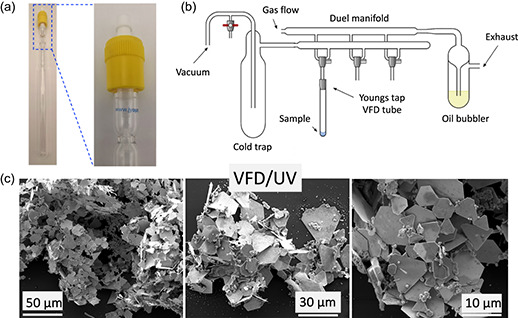
a) Photograph of a 20 mm OD quartz tube fit with a Young's tap. b) Schlenk line apparatus for preparing nitrogen atmosphere in a sealed tube. c) SEM images of gold nanosheets formed after 60 min VFD processing with *ω* = 5 k rpm, *θ* = 45°, 1 mL of 3.7 mm auric acid, with UV irradiation, *λ* = 254 nm.

The dramatically different structures formed by simply varying the concentration of auric acid, for both confined mode and continuous flow modes of operation of the VFD suggests that there are competing reactions taking place. In an attempt to decouple any such reactions, we carried out the processing under an atmosphere of nitrogen, noting that oxygen can undergo CE as a redox couple with oxidation of water to form H_2_O^+•^ which can lose H^+^ and give the hydroxyl radical, OH^•^.^[^ ^28^ ^]^ Nitrogen gas was first delivered through a jet feed into the VFD tube containing the auric acid solution which was then irradiated with UV at 254 nm, as shown in Figure S9, Supporting Information. VFD processing in the confined mode for 60 min with *ω* = 5 k rpm, *θ* = 45°, and 3.7 mm auric acid, under UV irradiation, *λ* = 254 nm, resulted in a high yield synthesis of 2D gold sheets with well‐defined larger structures relative to those formed in air, as shown in Figure S9, Supporting Information. Without UV irradiation in the VFD, a mixture of small amounts of 2D material and spherical particles resulted, as shown in Figure S9, Supporting Information. To ensure complete exclusion of oxygen during the processing, the VFD tube was fitted with a Teflon tap (Young's tap) and loaded with auric acid under nitrogen using a Schlenk line involving three cycles of freeze thaw, as shown in **Figure** **6**a,b. VFD processing (*ω* = 5 k rpm, *θ* = 45°, 60 min, 1 mL, 3.7 mm auric acid, confined mode, UV irradiation, *λ* = 254 nm) resulted in the formation of uniform 2D gold sheets, with the yellow color of gold discharged, and thus here the reduction of gold is essentially quantitative Figure 6c, unlike processing in air, and very little gold particles formed during UV irradiation of an auric acid solution without VFD processing and VFD processing without UV, as shown in Figure S10, Supporting Information. This is consistent with direct coupling of the photoinduced oxidation of water and reduction of auric acid, and that in air the reduction of O_2_ in forming O_2_^−•^ competes against the reduction of auric acid coupling with the oxidation of water. The superoxide radical anion itself can reduce auric acid in forming gold or generate hydrogen which reduces some auric acid, both affording singlet oxygen, Figure 2. Consistent with this is a drop in pH from 1.8 to 1.5 for processing under air, whereas under nitrogen it is reduced from 1.8 to 1.4.

### 2.4 Mechanistic Studies

#### 2.4.1 Photocontact Electrification

Under air and UV irradiation, the overall results are consistent with the formation of OH^•^ via proton loss of oxidised water, H_2_O^+•^, coupled with reduction of oxygen to the superoxide radical anion, O_2_^•−^,^[^ ^42^ ^]^ which can form hydroperoxyl, HO_2_^•^, leading to the formation of hydroxyl radicals by a chain reaction.^[^ ^28^ ^]^ The smoothing of gold surfaces can occur in the presence of OH^•^ radicals,^[^ ^37^ ^]^ and this may occur for long processing times, resulting in removing the divots on the gold prisms. The fate of O_2_^−•^ can be forming H_2_, Figure 2, and while hydrogen can reduce gold, even in the absence of UV irradiation, Figure S11, Supporting Information, the process is slow and any H_2_ removed from the system diminishes the amount of gold that can be formed for a certain processing time. This is reflected in the change in pH for processing under air, versus processing under nitrogen, as discussed above.

A competing reaction is the direct reduction of auric acid in a CE couple with photoinduced oxidation of water. Any gold oxide (Au_2_O_3_) formed by hydroxyl radicals would decompose under the localized high‐temperature topological fluid flows in the VFD,^[^ ^18^ ^]^ noting that gold oxide decomposes ≥160 °C and that elemental bismuth (m.p. 271.4 °C) melts in situ under ambient conditions in the VFD at the base of the ST flow.^[^ ^18^ ^]^ Hydroxyl radicals can also combine to form H_2_O_2_, as shown in Figure 2,^[^ ^43^ ^]^ which can then reduce the auric acid,^[^ ^44^ ^]^ with elemental gold catalytically decomposing H_2_O_2_ to water and oxygen.^[^ ^45^ ^]^ The superoxide radical anion can form singlet oxygen, ^1^O_2_, on reduction of auric acid. Reduction of auric acid by any H^•^ radicals and H_2_ is possible in forming elemental gold, noting that processing an auric acid solution in the VFD under hydrogen gas results in the formation of elemental gold as irregular 2D sheets, albeit in lower conversion, Figure S11, Supporting Information. The presence of either air or nitrogen atmosphere gas in the absence of UV irradiation VFD dramatically diminishes the formation of 2D gold.

#### 2.4.2 Processing Water in the VFD

Control experiments involved processing milli‐Q water only in the VFD at 5 k rpm for 60 min, *θ* = 45°, in air, with and without UV irradiation followed by adding 3.7 mm auric acid. After one hour, SEM images of the drop‐cast solution showed the prevalence of gold spherical nanoparticles for the water irradiated with UV in the VFD, with very little gold particles present for the same processing without UV irradiation, **Figure** **7**a and S15a,b, Supporting Information. This establishes the formation of reducing agents prior to adding the auric acid, possibly involving photo‐CE for the redox couple of water being oxidised to H_2_O^+•^ and O_2_ forming O_2_^−•^, which can generate hydrogen peroxide and hydrogen, Figure 2, 7f, and S17–18, Supporting Information. To gain further insight into the mechanism of formation of gold nanoparticles in the VFD, other studies were undertaken with the presence of ^1^O_2_, OH^•^, H_2_O_2_, and H_2_ confirmed/detected, as detailed later, Figure 7 and S15–18, Supporting Information.

Figure 7Redox couple reaction of water in the VFD. a) SEM images of gold nanoparticles formed after adding 3.7 mm auric acid to water previously treated in the VFD at 5 k rpm for 60 mins, *θ* = 45°, i) with and ii) without UV irradiation of the VFD tube. b) Singlet‐oxygen ^1^O_2_ test using 1,3‐diphenylisobenzofuran (DPBF) with a concentration of ratio 1:1 (0.6 mL of a DPBF solution with 0.6 mL of Milli‐Q water). i) UV−vis absorbance spectra of an aqueous solution of DPBF, and ii) picture of the DPBF color solutions for samples before VFD, VFD with UV irradiation, VFD only, and UV without VFD processing under air. c) ROS test using profuorescent nitroxide (PFN‐5) with concentration of ratio of 1:1 (0.6 mL PFN‐5 solution with 0.6 mL of Milli‐Q water). i) UV−vis absorbance spectra of an aqueous solution of PFN‐5, and ii) picture of PFN‐5 solutions before VFD, VFD with UV irradiation, VFD only, and UV without VFD processing under air. The VFD parameters for the (b,c) experiments were as follows: 5 k rpm, 60 min, with the VFD tube in the confined mode in air, with and without UV (254 nm) irradiation. d) Methylene blue test strips (40 mm × 60 mm) following adding 10 μL of processed milli‐Q water; the VFD processing was as follows: *ω* = 7.5 k rpm, *θ* = 45°, 20 min, 1 mL, confined mode, with fading of the blue color for liquid processed under UV associated with formation of colorless leuco‐methylene blue. e) ^1^H NMR of benzoic acid, BA (9 mm), processed under the same conditions, with and without UV irradiation. ^1^H NMR spectra of BA (9.0 mm) in water, processed under the same conditions (with and without UV, in air), in DMSO‐D_6_ using a 600 MHz NMR spectrometer, establishing the formation of isomeric hydroxybenzoic acids; higher UV irradiation (*λ* = 254 nm) concentrations of BA (36 mm) and for *θ* = 0° there was no NMR evidence for the formation of hydroxylated BA. f) Quantification of H_2_O_2_ generated during VFD processing using the potassium titanium oxalate (PTO) method.^[^ ^27^ ^]^ All reactions were *ω* = 7.5 k rpm and 5 k rpm, *θ* = 45°, 5 min, 2 mL, confined mode, under air or nitrogen atmosphere, with and without UV (254 nm) irradiation. The UV no VFD experiment was prepared using a 20 mm quartz tube at 0°, *θ* = 0 rpm. b) Concentrations of H_2_O_2_ 50−000 μm with all results a mean of triplicates.

**Figure:**
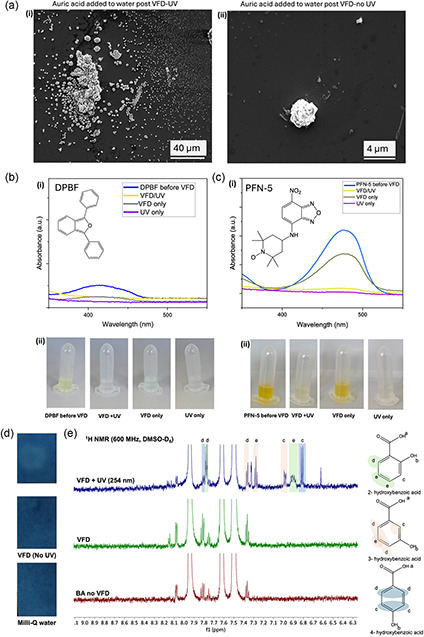

**Figure:**
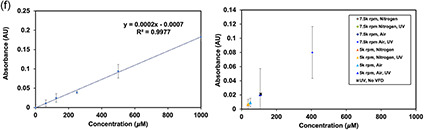

#### 2.4.3 Testing for Reactive Oxygen Species (ROS) and Hydrogen Gas

##### Singlet‐Oxygen Detection

1,3‐Diphenylisobenzofuran (DPBF), Figure 7b, is a nonfluorescent molecule that rapidly reacts with ^1^O_2_ via a [4 + 2] cycloaddition process, affording an endoperoxide product, with a loss of absorbance around 410‐430 nm.^[^ ^46^ ^]^ A solution of DPBF (3.1 mg) was prepared in EtOH (100 mL), for VFD processing, a concentration of 1:1 (0.6 mL of a DPBF solution with 0.6 mL of Milli‐Q water) was processed at 5 k rpm for 60 mins, resulting in a drop in intensity of the peak. Probe color changed by ≈50%, compared to the pre‐VFD solution, Figure 7b, confirming that singlet oxygen is generated. Processing the same reaction with UV for the same time resulted in essentially complete loss of the aforementioned peak and complete quenching of the yellow color, Figure 7b (ii), the same as with UV treatment alone (no VFD). As expected, for VFD processing under nitrogen, there was no change in UV spectra and no probe color changed was observed for UV irradiation and photobleaching with UV irradiation, Figure S15c, Supporting Information. The DPBF experiments establish the presence of ^1^O_2_ from water as a photo‐CE process in the VFD.

##### Other ROS

Profluorescent nitroxides (PFNs) is another probe used to detect ROS,^[^ ^47^, ^48^, ^49^ ^]^ and for this purpose we used PFN‐5, as shown in Figure 7c. An aqueous solution of PFN‐5 was prepared in ethanol at 0.031 mg. mL^−1^, the solution was stirred for 15 min. For VFD processing, a PFN‐5 solution 0.6 mL with 0.6 mL of Milli‐Q water in air resulted in quenching the probe color from by ≈20%, compared to the pre‐VFD solution and decrease in the intensity of the absorption peak at 480 nm in UV spectra, as shown in Figure 7c (i–ii); processing the same reaction with UV for the same time resulted in essentially complete quenching of the colour by 90% and reduction in absorbance at 480 nm after 60 min of processing, similar as UV only without VFD. Under nitrogen for the same processing resulted in quenching the yellow color when using UV only and a small reduction in intensity of the absorption peak at 480 nm using VFD processing only, Figure S15d, Supporting Information. This finding supports the source of oxygen reactive species under air as coming from the generation of ROS such as H_2_O_2_ and some photobleaching under nitrogen. The PFN‐5 test probe experiments establish the presence of ROS such as H_2_O_2_ and/or O_2_^−•^, both of which are produced via photo‐CE in the VFD.

##### Hydroxyl Radicals

The methylene blue strip test is used for detecting OH^•^ with its presence resulting in quenching of the blue color.^[^ ^50^ ^]^ This occurred in the present study for 1 mL of water processed in the VFD for 20 min with *ω* at 7.5 k rpm, under air while UV irradiated (*λ* = 254 nm), Figure 7d. A control of milli‐Q water was compared to processing 1 mL in the VFD for 20 min, *ω* 7.5 k rpm, with and without UV. A sample of the solution was dropped drop wise onto the center of the dyed blue section of the test strips, upon which the immediate and lasting bleaching occurred, confirming the presence of OH^•^, Figure 7d.

Hydroxylation of BA occurred in water in the VFD only while irradiated at 254 nm, as established using ^1^H NMR, and with no evidence for hydroxylation for processing under the same conditions, under an atmosphere of nitrogen, as shown in Figure 7e and S16, Supporting Information. The ^1^H NMR spectra are in good agreement with the expected peaks for each *o*‐, *m*‐, and *p*‐ substituted BA.^[^ ^51^ ^]^ Control experiments with the VFD at *θ* = 0° did not generate measurable quantities of hydroxylated BA while UV irradiated, nor did increasing concentration of BA to 36 mm with *θ* = 45°, without UV irradiation Figure S16, Supporting Information. The BA experiments establish that both UV irradiation at 254 nm and air are required for VFD processing (7.5 k rpm) in generating ROS, OH^•^ and/or O_2_^−•^, both of which arise from the photo‐CE process.

##### Hydrogen Peroxide Detection

Quantification for H_2_O_2_ used the PTO method involving spectrophotometric analysis for an absorption peak at 390 nm.^[^ ^52^ ^]^ Processing was under air or nitrogen, VFD and no VFD, and UV and no UV (*λ* = 254 nm), Figure 7f and S17, Supporting Information. The highest concentration of hydrogen peroxide was for VFD processing at *ω* = 7.5 k rpm under an atmosphere of air while UV irradiated. The rotational speed of 5 k rpm showed no increase in hydrogen peroxide compared to a control of irradiating the VFD tube with *θ* = 0°, *ω* = 0°. The potential to generate hydrogen peroxide using CE is noteworthy, in contrast to conventional photoelectrochemical and photocatalytic methods.

### 2.5 Hydrogen Gas Detection

For the photo‐CE involving the redox couple of water being oxidised to H_2_O^+•^ and O_2_ forming O_2_^−•^, hydrogen gas is assumed to form along with hydrogen peroxide. We tested the formation of hydrogen in situ in the VFD using a flammable gas detector with a flexible goose neck probe, as shown in Figure S18, Supporting Information. Water was processed for 5 min in air with the probe close to the open end of the VFD tube rotating at 5 k rpm, confined mode of operation, with and without UV irradiation (*λ* = 254 nm), each giving between 200 and 300 ppm of hydrogen gas. Placing the water in an open beaker immediately after VFD processing also gave a positive test when the probe was close to the surface of the water, for water treated in situ with UV and with UV irradiation. The same also occurred after processing 1 mL of water under nitrogen in a sealed tube fitted with a Young's tape. For comparison, aqueous auric acid solutions processed in the VFD for 5 min under air, with and without UV present, gave 400−500 ppm of hydrogen, and processing under nitrogen in a sealed tube gave 2000 ppm hydrogen gas.

### 2.6 Surface Modification Enhancement of Photo‐CE Effects

**Figure 8:**
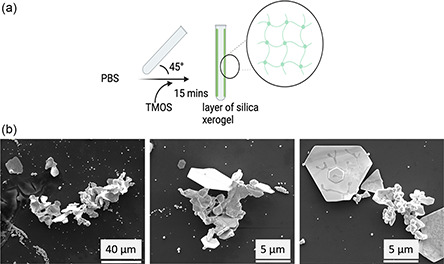
Enhancement of photo‐CE in the VFD. a) Schematic of coating the VFD tube (20 mm OD, 17.5 mm ID, 18.5 cm in length) with a layer of silica xerogel and b) SEM images for the resulting 2D gold formed in air VFD under UV irradiation, VFD parameters as follows: *ω* = 5 k rpm, *θ* = 45°, 60 mins, 1 mL, confined mode, *λ* 254 nm.

We hypothesized that the photo‐CE will be enhanced by increasing the surface area of the tube when it is coated with xerogel silica, as shown in **Figure** **8**.^[^ ^31^ ^]^ This was based on the ST topological flow having a much larger area of contact on the surface of the tube (≈1 μm in diameter) relative to DH flow (≈200 nm diameter),^[^ ^18^ ^]^ with ST resulting in much higher mass transfer into any porous material on the surface of the tube^[^ ^19^ ^]^ thereby potentially increasing S−L CE. In this context, xerogel surface‐type materials and porous membranes inserts on the surface of the VFD tube provide a matrix for incorporating catalysts, including enzymes, with increased catalyst loading relative to tethering the catalyst directly to the surface of the tube, for enhanced reaction outcomes while protecting the catalysts, as established for Pd nanoparticles embedded in cellulose membranes for selective hydrogenation under ambient conditions,^[^ ^53^ ^]^ laccase/copper phosphate nanoflowers embedded in xerogel silica,^[^ ^31^ ^]^ and copper phosphate magnetite composites, resulting in dramatically increased reaction rates.^[^ ^54^ ^]^ Herein, the inner surface of the VFD tube was coated with a layer of silica xerogel with processing in the confined mode under UV irradiation (*λ* = 254 nm), in air, at 5 k rpm. This resulted in complete discharge of the yellow color of aqueous auric acid solution, Figure S19, Supporting Information, and a high yield of elemental gold, mainly as 2D material, Figure 8. The is consistent with the xerogel coating increasing the interfacial surface area at the tube liquid interface^[^ ^31^ ^]^ enhancing photo‐CE. The uneven nature of the coating is likely to disturb the formation of Faraday waves and their DH flow. There is less reduction of auric acid for an uncoated quartz tube under identical processing conditions, including under air. Moreover, the higher mass transfer of auric acid into the silica xerogel layer by ST topological fluid is more likely to be rapidly reduced, either by OH^•^ and H_2_O_2_ from the oxidation of water and O_2_^•−^ from molecular oxygen or direct reduction via CE as a redox couple with the oxidation of water, as shown in Figure 2. Processing the same reaction but in the absence of UV resulted in little gold being formed, as shown in Figure S19c, Supporting Information. Carrying the reaction under a nitrogen atmosphere resulted in the xerogel turning pink, which is consistent with nanoparticles of gold trapped inside the gel, as shown in Figure S19d, Supporting Information. Reaction under nitrogen in the absence of UV has a similar effect to VFD processing under air without UV, as shown in Figure S19c,e, Supporting Information.

We have established VFD‐induced photo‐CE in water which can be further enhanced using xerogel silica coating the surface of the tube. This is a novel way for producing hydrogen and hydrogen peroxide from water and a novel way for preparing gold nanoparticles, with high uniformity of the product without the use of auxiliary chemicals with the processing scalable under continuous flow conditions, beyond what is possible using established methods,^[^ ^11^, ^12^, ^13^, ^14^ ^]^ with a comparison shown in **Table** **1**. VFD‐induced photo‐CE formation of gold nanoparticles also minimizes the waste generation on the processing and is without the need for heating and the use of high pressures, although such conditions prevail in the localized short‐lived submicrometer topological fluid flows in the VFD.^[^ ^17^, ^18^ ^]^

**Table 1:** Comparative table of the methods for preparing gold nanoparticles using VFD‐photo‐CE compared to other methods using plasma, microwave, and sonication energy sources.

Energy source	Plasma‐induced electrification (PIE)^[^ ^14^ ^]^	VFD‐induced CE (This study)	Microwave^[^ ^12^ ^]^	Sonication^[^ ^13^ ^]^
Environmental Impact	Minimizes chemical usage and waste generation	No chemicals; no waste	May requires chemicals	May requires chemicals
Scalability	Scalable	Scalable	Scalable	Scalable
Efficiency	Rapid and precise	High and continuous	Variable	Variable
Process Control	Precise parameter control	Consistent parameters	Limited control	Limited control
Chemical Requirements	Involves precursor chemicals	Only water needed	May involve chemicals	May involve chemicals
Heat and Pressure Requirements	May need elevated conditions	No heat or pressure needed	Heat and pressure may be required	Heat and pressure may be required

## 3 Conclusion

Processing aqueous auric acid solutions in the VFD results in several competing reactions, depending on the concentration of auric acid and operating parameters of the device, UV irradiation, surface porosity, and the atmosphere inside the rotating tube, with water itself able to react under UV irradiation. Photo‐CE occurs on UV irradiation (*λ* = 254 nm) in air as a redox couple forming reactive species, OH^•^ and H_2_O_2_, from oxidation of water to H_2_O^+•^, and reduction of oxygen to O_2_^−•^, all resulting in the formation of gold nanoparticles, with O_2_^−•^ forming singlet oxygen. Nanoparticles are also formed by direct reduction of auric acid in a redox couple with the aforementioned oxidation of water. Under photo‐CE conditions in air, different nanogold materials are formed, notably 2D ultrathin sheets of gold, prisms of gold, and a hierarchical structure comprising gold prisms encapsulated within ultrathin sheets of gold and hollow tubes, and the ability to generate gold in a controlled way as such is without precedent.

The different shapes and dimensions of the nanogold are effectively molds of the topological fluid flows in the VFD which otherwise cannot be measured directly in a rotating reference frame. Photo‐CE occurs in pure water, affording OH^•^ and H_2_O_2_, and O_2_^−•^, which is quenched by water forming singlet oxygen, ^1^O_2_, and H^•^ enroute to H_2_. We find that hydrogen in the VFD also results in the formation of elemental gold, mainly as 2D gold. Under an atmosphere of nitrogen, photo‐CE now involves direct reduction of auric acid, but with all the gold reduced, unlike in air, forming 2D gold in essentially quantitative yields. 2D gold also forms under continuous flow conditions which augers well for applications of this unique form of free‐standing pristine gold devoid of surfactants.

## 4 Experimental Section

4.1

4.1.1

##### Materials

Auric acid (H[AuCl_4_]), 99.9% purity in aqueous HCl, phosphate‐buffered saline (PBS), tetramethoxysilane (TMOS), 1,3‐diphenylisobenzofuran (DPBF), ethanol, 1,3‐diphenylisobenzofuran (DPBF), nitroxide [55‐tetramethyl‐4‐[(7‐nitro‐ 2,1,3‐benzoxadiazol‐4‐yl)amino]‐1‐piperidinyl] oxidanyl radical (PFN‐5), BA, PTO (>99%), methanol, and DMSO‐d6 were all purchased from Sigma Aldrich.

##### Synthesis of Gold Nanoparticles

The as‐received aqueous auric acid was diluted in Milli Q‐water to obtain 3.0, 3.7, 4.0, 7.0, and 13.0 mm concentrations of the metal complex. The pH of the solutions before VFD processing was 2.0, 1.8, 1.7, 1.5, and 1.0, respectively. These concentrations of auric acid solutions were used directly in a VFD operating at room temperature, from ≈22 to 24 °C. For the confined mode of operation of the VFD, 1 mL of the diluted solution was placed in a 20 mm‐OD (17.5 mm ID) quartz tube, 18.5 cm in length, with a hemispherical base (unless otherwise stated) and open at the other end, essentially taking on the shape of a conventional test tube. The tilt angle (*θ*) of the tube was fixed at 45° unless otherwise stated with the tube spun at the specified rotational speed (*ω*), for a designated time. For continuous flow processing, the tube was also tilted at 45° with a flow rate of the same solution at 0.3 mL min^−1^ unless otherwise stated. For all processing in the VFD, the quartz tube was irradiated on both sides along the length of the tube, with UV‐light‐emitting diodes (LEDs) operating at ≈*λ* 254 nm, 20 W (except for experiments in the absence of UV irradiation), Figure 1 and S1, Supporting Information, for 60 min processing in the confined mode unless otherwise stated, and for a certain volume of liquid, as specified, or under continuous flow. The resulting solutions were centrifuged for 1 min at 6900 × *g* whereupon the resulting gold material was washed with Milli Q‐water (1 mL). Samples were then drop cast onto silicon wafers and left to dry in air for characterization purposes, as follows: SEM and EDX, XPS, scanning Auger nanoprobe, and AFM. For TEM, particles were dispersed on a standard grid, and XRD studies were for dry samples on silicon substrates. Further details are provided below and in the Supporting Information.

##### Xerogel Silica Hydrogel Coating

PBS prepared at pH 7.4 and tetramethoxysilnae (TMOS) were used without further purification. Stock solution of PBS was prepared with deionized water (Milli Q). 1 mL of PBS was added to a VFD tube followed by 495 μL of TMOS for 15 min processing at 5 k rpm, resulting in in situ gelation, as a modification of a published procedure^[^ ^31^ ^]^ with the addition of PBS accelerating the gelation process down to 15 min while processing at *ω* 5 k rpm. Thereafter, 1 mL of 3.7 mm auric acid solution was added to the VFD tube coated with the silica hydrogel along the entire length. The VFD parameters for the formation of gold using the xerogel silica hydrogel coating experiment were as follows: *ω* 5 k rpm for 60 min in air or nitrogen, with the tube capped and operated in the confined mode with and without UV irradiated at *λ* 254 nm.

##### Characterization

Samples of gold nanomaterial generated in the VFD were added to a silicon substrate as a colloidal suspension in MilliQ‐water by drop casting, followed by evaporation under ambient conditions. Morphologies, size, and shapes of the particles were studied using SEM (FEI F50) operating at an accelerating voltage of 10 kV with a 10 mm working distance and equipped with EDX capability. Size distribution plots were generated using image J software. Zeta potential distributions, ζ, of the gold nanoparticles were determined in water at 25 °C using a Nano Zetasizer from Malvern Instruments. Studies were undertaken using a PHI‐710 Scanning Auger Nanoprobe. Scanning electron micrographs, scanning Auger micrographs, and Auger electron spectra were obtained using an electron beam voltage of 10 k V, with a beam current of 10 nA and pressure in the analysis chamber during the analysis maintained below 10^−9^ Torr. AFM was used for acquiring information on force measurements, topographic imaging, and manipulation of the nanoparticles. AFM images were obtained in air using a Bruker Multimode 8 AFM with Nanoscope V controller, operating in standard tapping mode. The AFM probes used were silicon HQNSC15/AlBS Mikromasch probes (nominal tip diameter and spring constant of 16 nm and 40 N m^−1^ respectively). Set point, scan rate, and gain values were chosen to optimize image quality. AFM topography images were flattened and height measurements were made using the section analysis tool of Nanoscope Analysis. The AFM scanner was calibrated in *x*, *y*, and *z* directions using silicon calibration grids (Bruker model numbers PG: 1 μm pitch, 110 nm depth and VGRP: 10 μm pitch, 180 nm depth and Mikromasch model TGZ01: 3 μm pitch, 18 nm depth). XRD patterns were obtained using an X‐ray diffractometer (Bruker, Germany) using Co Kα radiation (*λ* = 1.78892 Å) with the 2*θ* scanning range from 10° to 90°. Brunauer–Emmett–Teller (BET) was used to measure the porosity. XPS instrument was provided by SPECS (Berlin) with a no‐monochromatic X‐ray source (12 kV–200 W) with Mg anode used for the measurements. The operation was performed under ultrahigh‐vacuum condition with a base pressure of e‐10 mbar. The samples were mounted on a Mo sample holder. Semiconductor‐grade Si was used as a substrate. The conductivity of Mo holder and Si substrate was sufficient for the electron compensation due to the X‐ray radiation and thus avoiding any charging of the samples. TEM was performed using a JEOL JEM‐F200 Multi‐Purpose FEG‐S/TEM operating at an accelerating voltage of 200 kV. Image J was used for processing the images.

##### Testing for Reactive Oxygen Species: Detection of ^1^O_2_

1,3‐Diphenylisobenzofuran (DPBF) is widely used for the detection of ^1^O_2_.^[^ ^46^ ^]^ All testing experiments involving DPBF were carried out in the dark, given that DPBF is sensitive to light. DPBF is not soluble in water, so a 1:1 mixture of ethanol and water was used; a fresh solution of DPBF (3.1 mg) in EtOH (100 mL) was stirred in the dark for 15 min whereupon 0.6 mL of a DPBF solution was added to the VFD along with 0.6 mL of Milli‐Q water. The VFD parameters for the experiments were as follows: 5 k rpm, 60 min, with the VFD tube capped and operated in the confined mode under air, with and without UV (*λ* = 254 nm) irradiation. UV−vis absorbance spectra were recorded immediately after processing with probe color change observation. For more control experiments, the DPBF solution with water was tested in the absence of VFD. For comparison, testing for the presence of ^1^O_2_ was carried out under nitrogen using a Youngs tape VFD tube, Figure 6a,b.

##### Testing for Reactive Oxygen Species: Other Reactive Oxygen Species

Nitroxide [55‐tetramethyl‐4‐[(7‐nitro‐ 2,1,3‐benzoxadiazol‐4‐yl)amino]‐1‐piperidinyl] oxidanyl radical (PFN‐5):^**[**^ ^47^ ^**]**^ Experiments involving PFN‐5 were similarly performed to that for DPBF. The concentration of PFN‐5 in ethanol was 0.031 mg.mL^−1^ with the solution stirred for 15 min. Thereupon, the PFN‐5 solution was mixed with water in the VFD, under air or nitrogen with and without UV irradiation (*λ* = 254 nm) and also in the absence of VFD processing. The VFD parameters for the experiments were as follows: *ω* = 5 k rpm, 60 min, with the tube capped and processed in the confined mode. The UV−vis absorbance spectra were recorded immediately after VFD processing with probe color change observation.

##### Testing for Reactive Oxygen Species: Hydroxyl Radical Detection (Methylene Blue Test Strip Method)

Test strips of filter paper were prepared by cutting 20 mm × 60 mm small rectangular strips (grade 1, medium porosity, Fisher scientific) following a previously outlined method.^[^ ^50^ ^]^ The dye section was prepared by measuring 1 cm from the base of the small edge of the strips and marking across with a fine black permanent marker. The strips were dipped 10 times successively into a 1.0 mm MB dye solution until the color reached the marker line. The test strips were then left to dry in a dark box for 24 h whereupon they were used for testing purposes. A control of milli‐Q water was compared to processing 1 mL in the VFD operating under confined mode for 20 min at *ω* = 7.5 k rpm with and without UV (254 nm) irradiation.

##### Testing for Reactive Oxygen Species: Hydroxylation of Benzoic Acid

Experiments used a solution of 1.2 mg of BA in 1 mL of Milli‐Q water with processing as follows: *ω* = 7.5 k rpm, 20 min, with the tube capped and processed in the confined mode. Postprocessing, a rotary evaporator was used to remove water with the solid residue dissolved in methanol for first thin‐layer chromatography (TLC) analysis before another rotary evaporation step and dissolution of the product into DMSO‐d6 for NMR studies. Three solutions were spotted at the bottom of the TLC plates: (1) 9 mm BA in MeOH, (2) 9 mm BA processed in VFD, and (3), as for (2), with UV (254 nm) and power of with UV‐LEDs, *λ* = 254 nm, 20 W. The eluting solvent was a 1:5 mixture of methanol and chloroform. After drying, the TLC plates were examined under UV (*λ* = 254 nm) and the spots outlined with a pencil. The retention factors (R_f_) were calculated as the distance travelled by the control BA/MeOH solution divided by the distance travelled by the sample. NMR spectroscopy used a NMR600 (PS240) instrument and DMSO‐D_6_ as a solvent. Chemical shifts were reported in ppm.

##### Testing for Reactive Oxygen Species: Hydrogen Peroxide Analysis

Quantification of Hydrogen Peroxide (H_2_O_2_) Formation used the PTO (K_2_TiO(C_2_O_4_)_2_.H_2_O) Method.^[^ ^52^ ^]^ This Involved Spectrophotometric Analysis: a 0.1 M PTO Solution Was Prepared and a Calibration Curve Determined for Concentrations Ranging from 0 to 1000 μm Hydrogen Peroxide Concentration in Milli‐Q Water Using Serial Dilution and Repeated in Triplicates. The *R* ^2^ Value Was in Good Agreement for a Linear Relationship Between Absorbance and Concentration (*R* ^2^ > 0.99). The VFD Parameters for the Experiments Were as Follows: 7.5 k rpm and 5 k rpm, 5 min, Using a Young's Tap VFD Tube to Maintain an Air or N_2_ Gas Environment, with and Without UV Irradiation (*λ* = 254 nm). following VFD Processing, 1 mL of the Sample Was Combined with 1 mL of the PTO Solution, and 200 μL Samples Were Prepared on a 96‐Well Plate. The Absorbance at 390 nm Was Determined through Measurements on a CLARIOstar Spectrophotometer

##### Hydrogen Detection

A digital gas sniffer, Model: JL269, flammable gas detector with a flexible goose neck probe was used to detect hydrogen gas (H_2_), as shown in Figure S18, Supporting Information. 1 mL of water and 1 mL of 3.7 mm auric acid were tested separately during processing in the VFD under the following parameters: ω = 5 k rpm, 5 min, hemispherical base VFD tube under air, or under nitrogen using a Young's tap VFD tube, with and without UV irradiation (*λ* = 254 nm), all giving a positive test for hydrogen evolution, as shown in Figure 6a,b. Each liquid was placed in an open beaker immediately after VFD processing which also gave a positive test when the probe was close to the surface of the liquid.

## Conflict of Interest

The authors declare no conflict of interest.

## Author Contributions

B.A. performed all the vortex fluidic device experiments, data analysis for scanning electron microscopy, energy‐dispersive X‐ray spectroscopy, X‐ray diffraction, Auger and X‐ray photoelectron spectroscopy, UV–visible spectroscopy, DPBF, PFN‐5 and xerogel studies, and H_2_ detection. Z.G. carried out the BA experiments, hydroxyl radical, and hydrogen peroxide detection. X.C. and T.M. carried out the transmission electron microscopy analysis. K.V. carried out the atomic force microscopy analysis. B.A. and C.L.R. developed the overall contact electrification mechanism. C.L.R. coordinated the research and developed the model for the fluid behavior. The primary content of the manuscript was written by B.A., and Z.G. wrote the BA and H_2_O_2_ contents following the completing of the first full draft by B.A. and C.L.R. All authors have given approval to the final version of the manuscript.

## Associated Content

The supporting information is available free of charge. Characterizations include SEM, TEM, XPS, AFM, UV‐Vis, XRD, and ^1^H NMR spectroscopy.

## Supporting information

Supplementary Material

## Data Availability

The data that support the findings of this study are available in the supplementary material of this article.
