# Supplementary material for: Nanogold Foundry Involving High‐Shear‐Mediated Photocontact Electrification in Water
Source: Small Sci. 2024 May 9;4(6):2300312. doi: 10.1002/smsc.202300312 (PMC11935251; doi:10.1002/smsc.202300312)
Supplement: Supplementary file 1 — Supplementary Material [file SMSC-4-2300312-s001.pdf]

Supplementary information

Nanogold foundry involving high shear mediated photo contact electrification in water

Badriah M. Alotaibi,[a] *Zoe Gardner,*[a] *Kasturi Vimalanathan,*[a] *Xianjue Chen,*[b] *Thaar M. D. Alharbi,*[a,c] *Colin L. Raston*[*a]

Table of Contents

Nanogold foundry involving high shear mediated photo contact electrification in water *1*

1. Abstract *3*

2. Experimental methods *4*

2.1. Vortex Fluidic Device (VFD) 4

3. Synthesis of 2D-sheets *4*

Gold synthesis - Optimised 2D gold conditions 4

3.1. VFD/UV Present Effect 6

3.2. Changing Reaction Times 7

3.3. Changing Rotational Speeds 8

3.4. Changing Concentrations 9

3.6. Changing Flow Rate - Continuous Flow 11

3.7. Changing Reaction Atmosphere- Under N_2 Using a Youngs Tape 13

3.8. VFD processing under a hydrogen atmosphere 14

4. Additional Characterisations For 2D Gold Nano Sheet *15*

5. Other Method- Sonication *17*

6. Testing For Reactive Oxygen Species (ROS) *18*

7. Hydrogen Detection *22*

8. Enhancement of CE in the VFD *23*

Coated The Tube with a Layer of Silica Xerogel in the formation of nano gold 23

9. Author Contributions *24*

1. Abstract

Controlling the size and morphology of gold nanoparticles occurs in the absence of added reducing agents or other excipients such as surfactants, on UV irradiation (λ 254 nm) of aqueous auric acid ($H[AuCl_4]$) in a thin film of liquid generated in a vortex fluidic device (VFD) within a rapidly rotating tilted quartz tube. This involves contact electrification (CE) which occurs at the solid-liquid interface with the oxidation of water photo-induced, forming the hydroxyl radical, $OH^{\bullet}$. In air, the redox couple is reduction of $^3O_2$ to the superoxide radical anion, $O_2^{-\bullet}$, which then reduces Au^{3+} to elemental gold, as does other reactive oxygen species present, competing with CE reduction of Au^{3+}. The resulting nanogold structures effectively mould the different high shear topological fluid flows in the VFD, being isolated as ultra-thin two-dimensional (2D) sheets, prisms, hierarchical structures comprised of nanoparticles embedded within these sheets, and rosette and tubular structures, depending on the VFD processing parameters and the concentration of auric acid. Processing under a nitrogen atmosphere while similarly UV irradiated affords mainly 2D gold through the above reduction of Au^{3+}. The findings establish a paradigm for VFD processing in water under such UV irradiation involving photo-induced CE, generating hydrogen peroxide in situ and hydrogen gas. Gold nanomaterials are accessible beyond what is possible using traditional processing strategies, with the processing high in green chemistry metrics and the surfaces of the gold nanoparticles are pristine.

2. Experimental methods

2.1. Vortex Fluidic Device (VFD)

The VFD has two different processing modes, confined mode, and continuous flow. The feasibility of scaling up into continuous flow processing, was explored where the solution is continuously fed down the tube through jet feeds protruding the Teflon housing.

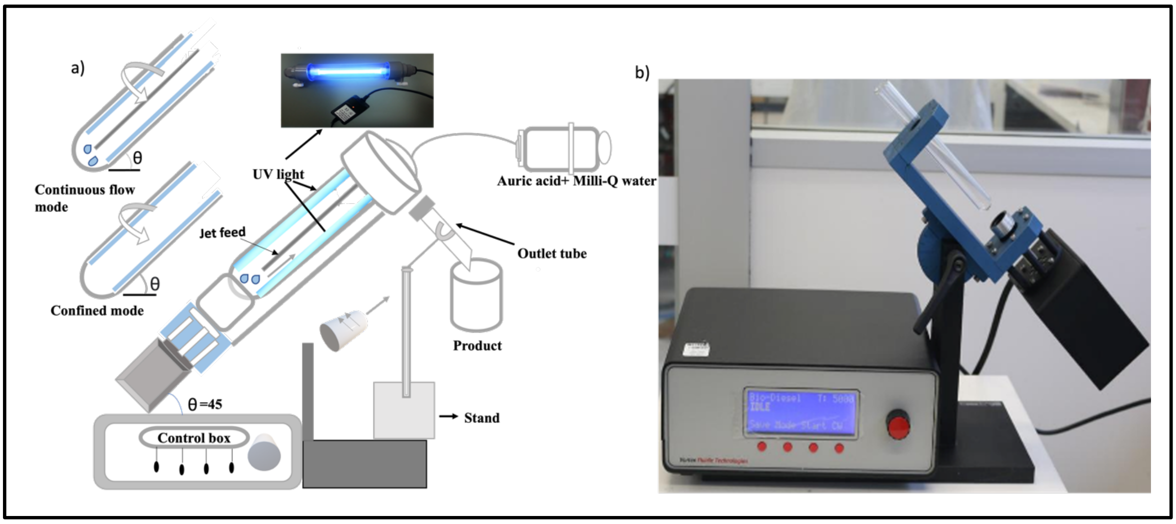

Figure S1: (a) Schematic illustration of the overall process for the synthesis of gold nanostructures under shear stress in a vortex fluidic device (VFD) photographed in (b) using hemispherical base of the quartz tube, 20 mm O.D. (17.5 mm I.D.), 18.5 cm long. The optimal VFD processing condition for the formation of 2D gold was as follows: speed ω = 5000 rpm, θ = 45˚, time (t) = 60 mins, concentration c= 3.7 mM, volume (V) = 1 mL for VFD processing using the confined mode, and flow rate ($\dot{v}$) = 0.2 and 0.3 mL.min^{-1} for using continuous flow mode.

3. Synthesis of 2D-sheets

Gold synthesis - Optimised 2D gold conditions

The as received auric acid ($H[AuCl_4]$) was diluted in Milli Q-water to obtain 3, 3.7, 4-, 7- and 13-mM concentrations of gold solution. The 3.7 mM concentration was chosen as optimal concentration as it is providing a well-defined triangle and hexagonal 2D structures. The pH was chosen to be low as higher pH afforded thick gold structures, Figure S7(b). The auric acid solutions were used directly in a VFD at room temperature in a 20 mm diameter (17.5 mm internal diameter) quartz tube, 18.5 cm in length, with a hemispherical base and open at the other end, essentially taking on the shape of a conventional test tube. Changing the base shape from hemispherical base to flat base perturbs the Coriolis flow arises from the bottom of the tube, now having a much smaller radius of curvature which is limited by the fabrication of the

quartz tube itself. For the flat based tube there was no evidenced for the formation of the 2D material, Figure S7(a).

The tilt angle θ of the tube was fixed at 45° as optimal tilt angle. There is no 2D structures formed at 0 degree, Figure S7(c). The optimal rotational speed (ω) was 5k rpm relative to 4k and 7k rpm; processing gold solution at 4k rpm resulting of rod structures while 7k rpm resulted in prisms of gold structures, Figure S4,5. Time was optimised to be 60 mins for confined mode with 3, 7 and 30 min processing providing insight into the growth of the particles into 2D structures, Figure S3. The optimised flow rates for 2D gold structure in the continuous flow processing were 0.2 and 0.3 $mL.min^{-1}$ Figure S8(b), even though the high flow rates of 0.4 and 0.5 $mL.min^{-1}$ gives gold material but as less defined 2D structures Figure S8(a). For all processing in the VFD, quartz tubes were irradiated on both sides along the length of the tube, with UV-LEDs operating at approximately (λ 254 nm), 20 W; without UV irradiation, only small amounts of nanogold formed. The resulting solutions were centrifuged for 1 minutes at 6900 x g whereupon the resulting gold material was washed with Milli Q-water (1 mL). Samples were then dropping cast onto silicon wafers and left dry in the air for different characterisation. Approximately 60% of the auric acid was reduced to gold material for processing under air, as determined using SEM. Changing the atmosphere to nitrogen whilst keeping the rotational speed, and concentration of auric acid the same, complete discharge of the yellow colour arising from auric acid occurred, with the isolated yield of 2D material ~ 90%.

The conditions of rotational speed, atmosphere, tube base and VFD angle were optimised, as discussed above. The conditions were optimised according to the highest yield and well-defined 2D structures, as evident from SEM after drop casting on a silicon wafer, and the formation of the most consistent shapes and sizes. A rotational speed of 5k rpm was chosen as optimal, which resulted in the largest 2D sheets and formed in larger amounts, relative to the other rotational speeds.

3.1. VFD/UV Present Effect

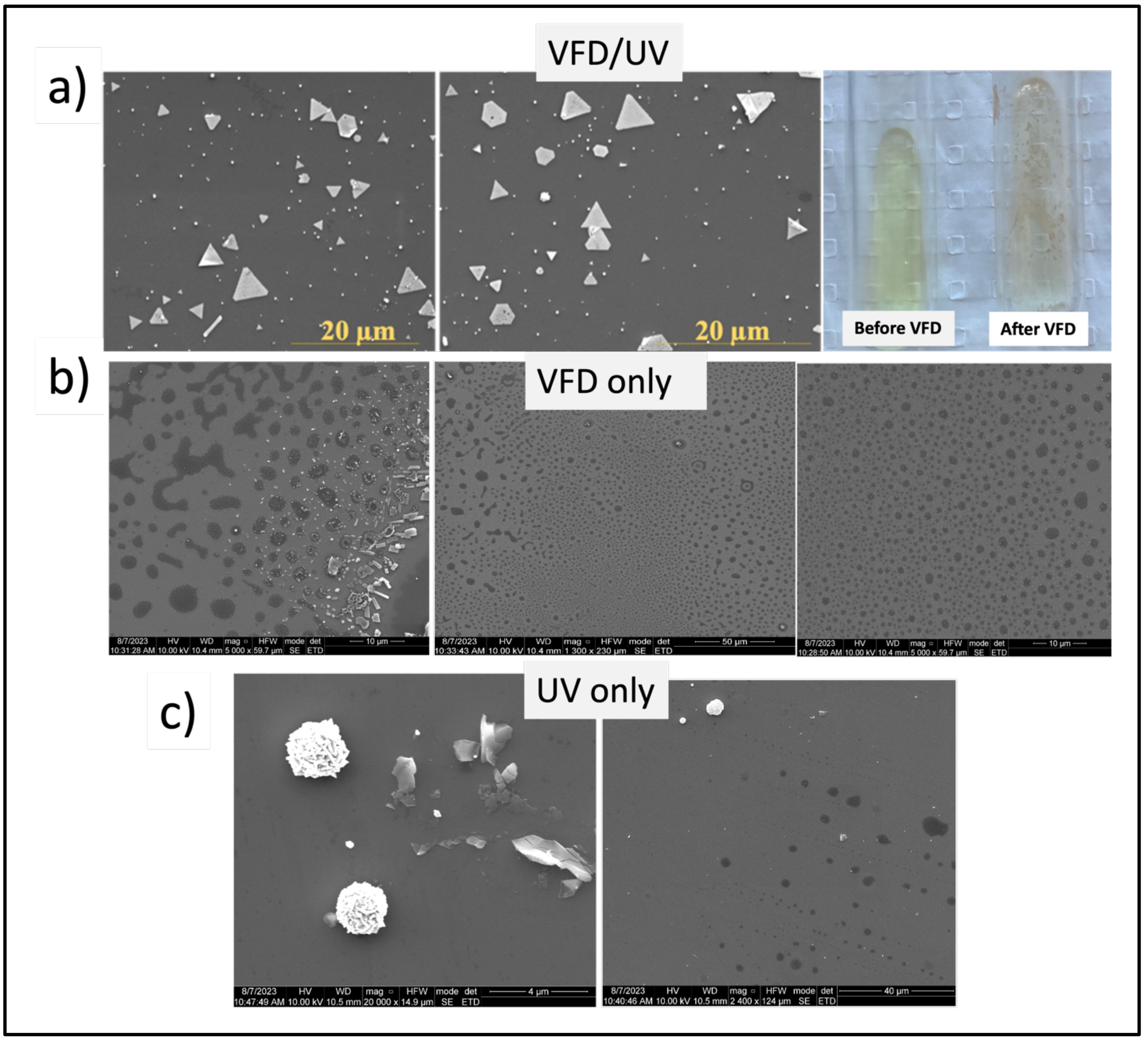

Figure S2: SEM images of gold nano particles formed after VFD processing in *air atmosphere* with different control experiments, VFD/UV, where solid of gold formed after VFD as pictured(a), VFD only (b)and UV only (c). VFD processing was as follows: speed ω = 5000 rpm, θ = 45°, time 60 mins, volume = 1 mL, concentration (c) = 3.7 mM, VFD processing in the confined mode, (λ = 254 nm).

3.2. Changing Reaction Times

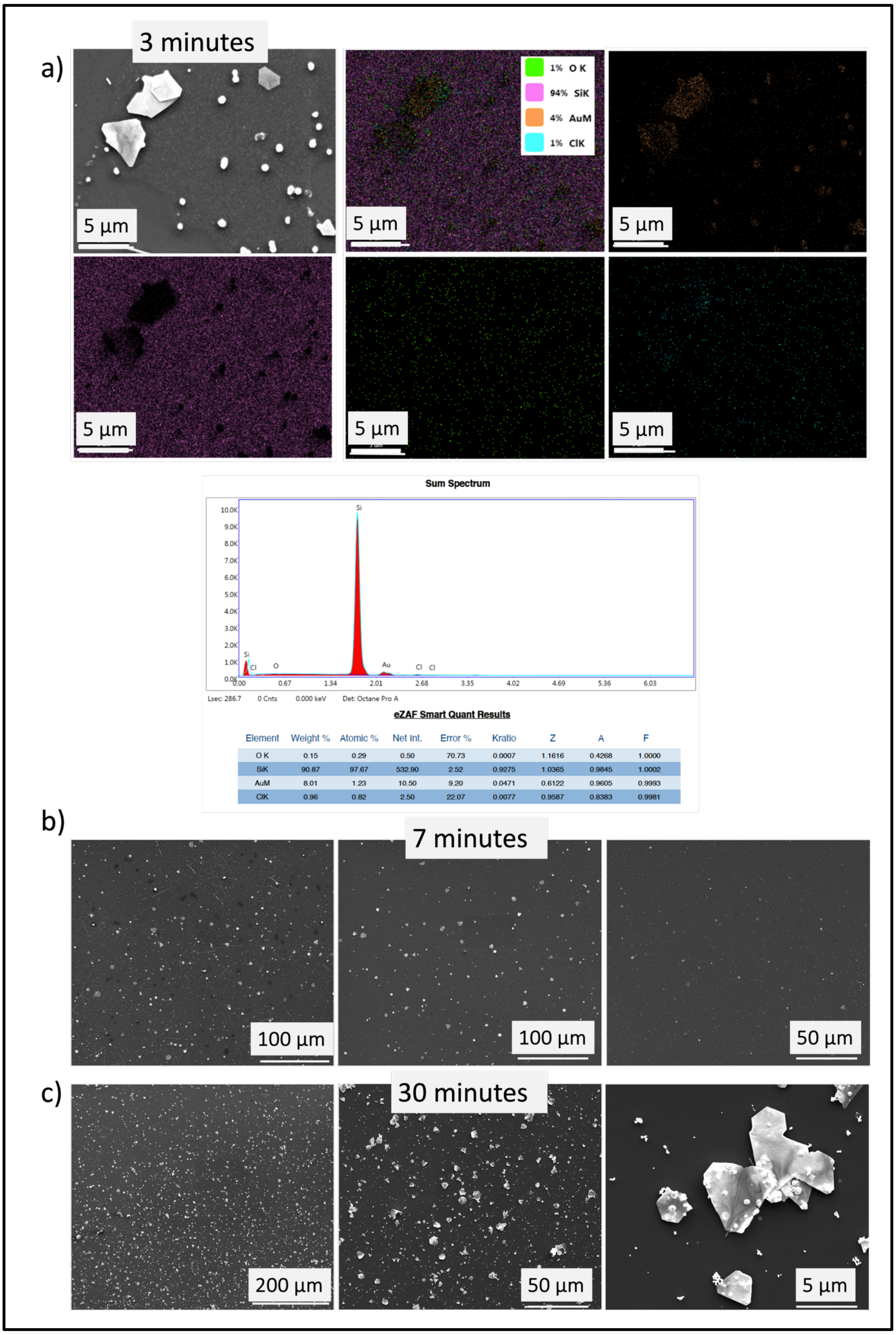

Element	Weight %	Atomic %	Net Int.	Error %	Kratio	Z	A	F
O K	0.15	0.29	0.50	70.73	0.0007	1.1616	0.4268	1.0000
SiK	90.87	97.67	532.90	2.52	0.9275	1.0365	0.9845	1.0002
AuM	8.01	1.23	10.50	9.20	0.0471	0.6122	0.9605	0.9993
ClK	0.96	0.82	2.50	22.07	0.0077	0.9587	0.8383	0.9981

Figure S3: SEM/EDX images of gold nano particles formed after VFD processing with *different time* processing for (a) 3 min, as indicated in EDX (b) 7 min, (c) 30 min with concentration, 3.7 mM. The VFD processing was as follows: speed ω = 5K rpm, θ = 45˚, volume = 1 mL, confined mode, (λ = 254 nm).

3.3. Changing Rotational Speeds

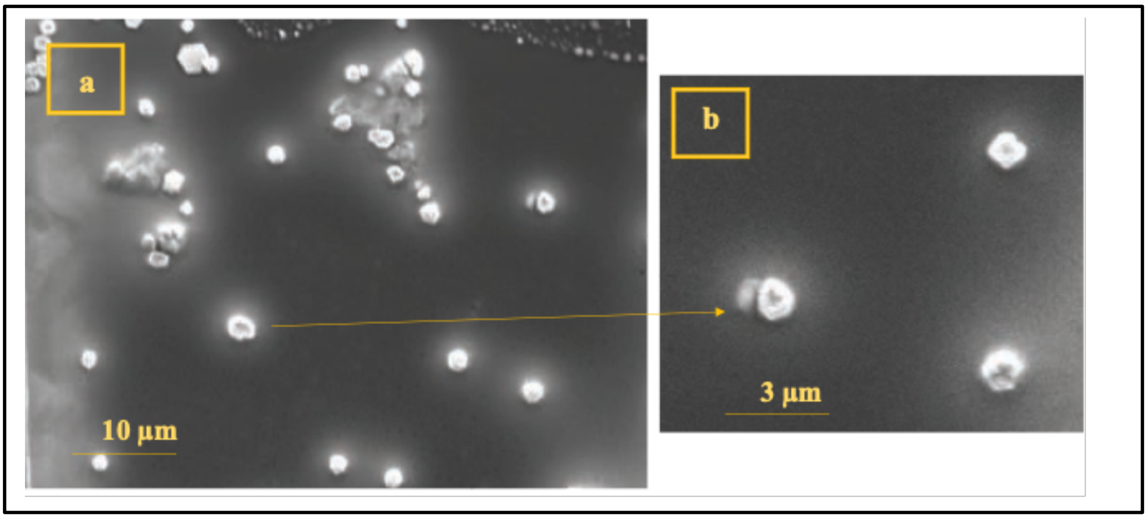

Figure S4. SEM images of gold nano particles formed after VFD processing resulted in a prism of gold with holes *at 7k rpm rotational speed,* with concentration, 3.7 mM. The VFD processing was as follows: speed ω = 7k rpm, θ = 45˚, time = 60 mins, volume = 1 mL, confined mode, (λ = 254 nm).

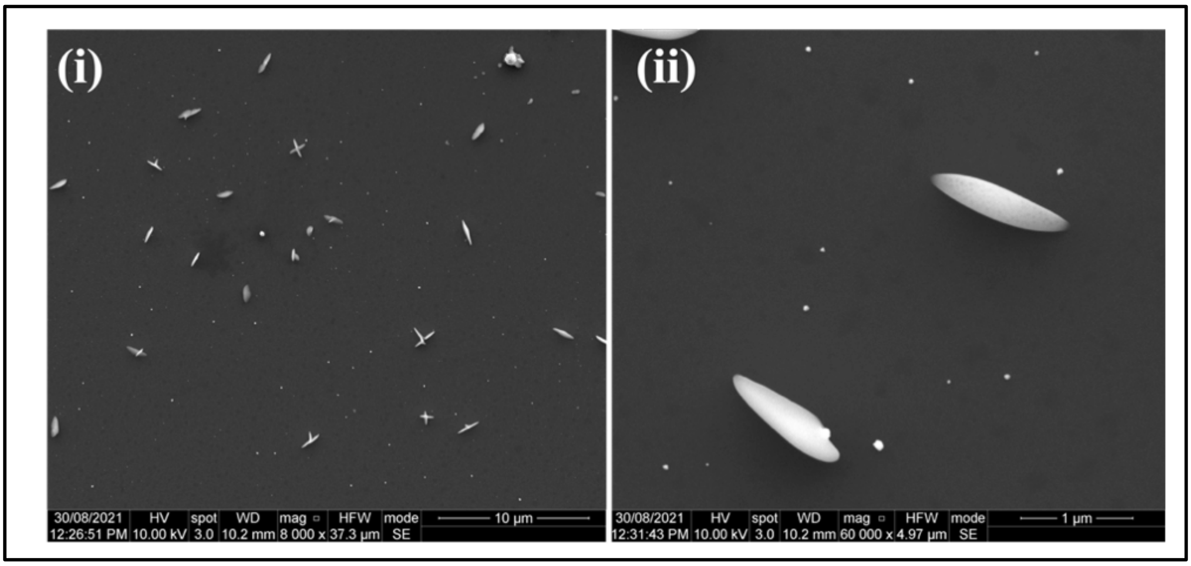

Figure S5: SEM images of gold nano particles formed after VFD processing resulted in rods of gold formed *at 4k rpm rotational speed*, with concentration, 3.7 mM. The VFD processing was as follows: speed ω = 4K rpm, θ = 45˚, time = 60 mins, volume = 1 mL, confined mode, (λ = 254 nm).

3.4. Changing Concentrations

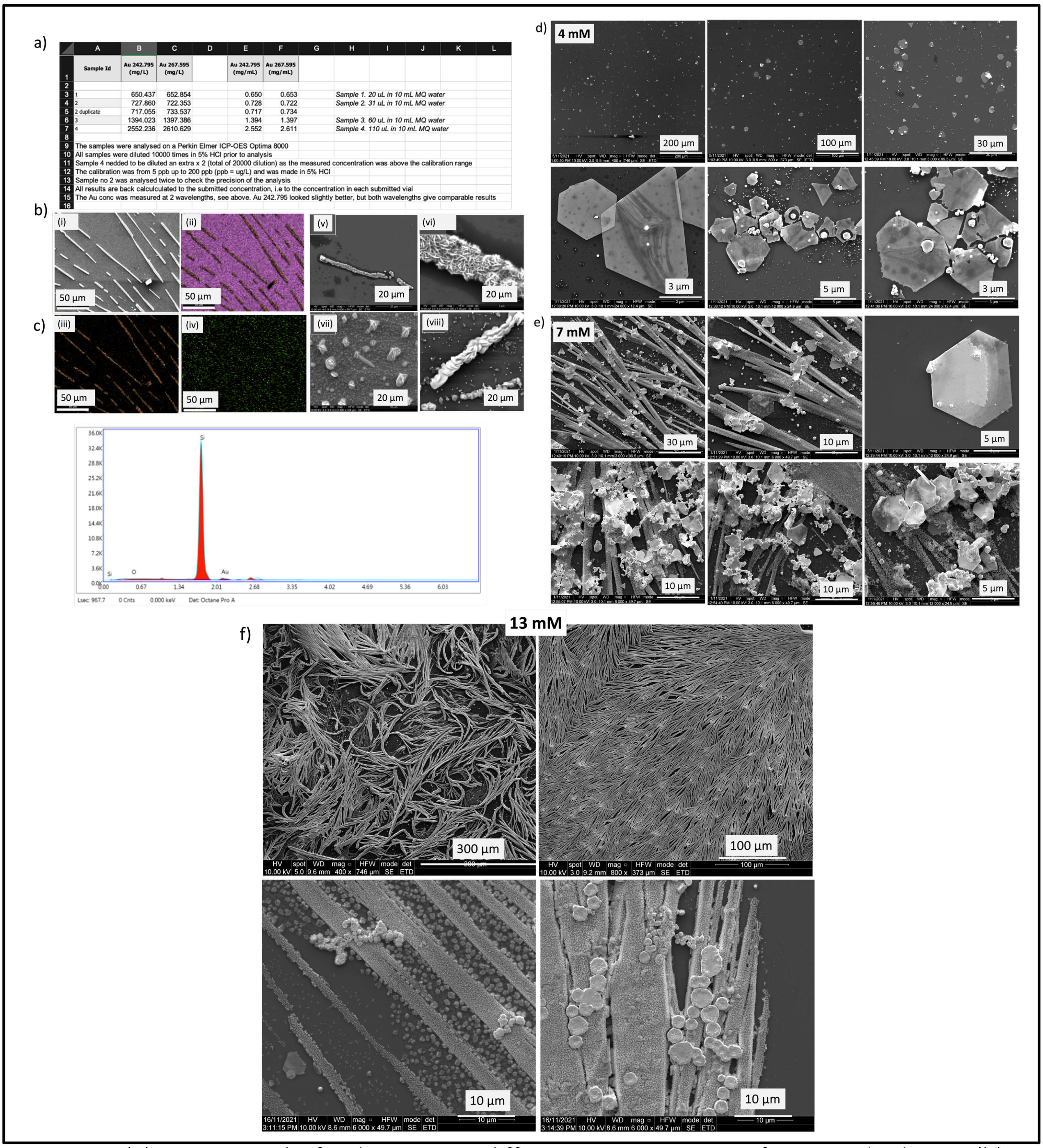

Figure S6. (a) ICP-MS results for determining different concentrations of auric acid solution. (b) SEM images of hollow tubes gold and hollow tube-like structure of gold formed under low concentration, 3 mM (b), and (b-c) (i-iv) SEM/EDX gold nano tubes, resulting in instability of the particles, undergoing Oswald ripening over time (v-viii). (d-f) SEM images for higher concentrations of gold, 4, 7 and 13 mM, respectively, affording 2D material at 4 mM and with higher concentrations, the 2D structure regrew into bigger structure making a composition of ultrathin sheets of gold encapsulating rosette shaped nanostructures. The VFD processing was as follows: speed ω = 5K rpm, θ = 45˚, time = 60 mins, volume = 1 mL, confined mode, (λ = 254 nm).

3.5. Changing VFD Base Tube – pH – VFD Tilt Angle

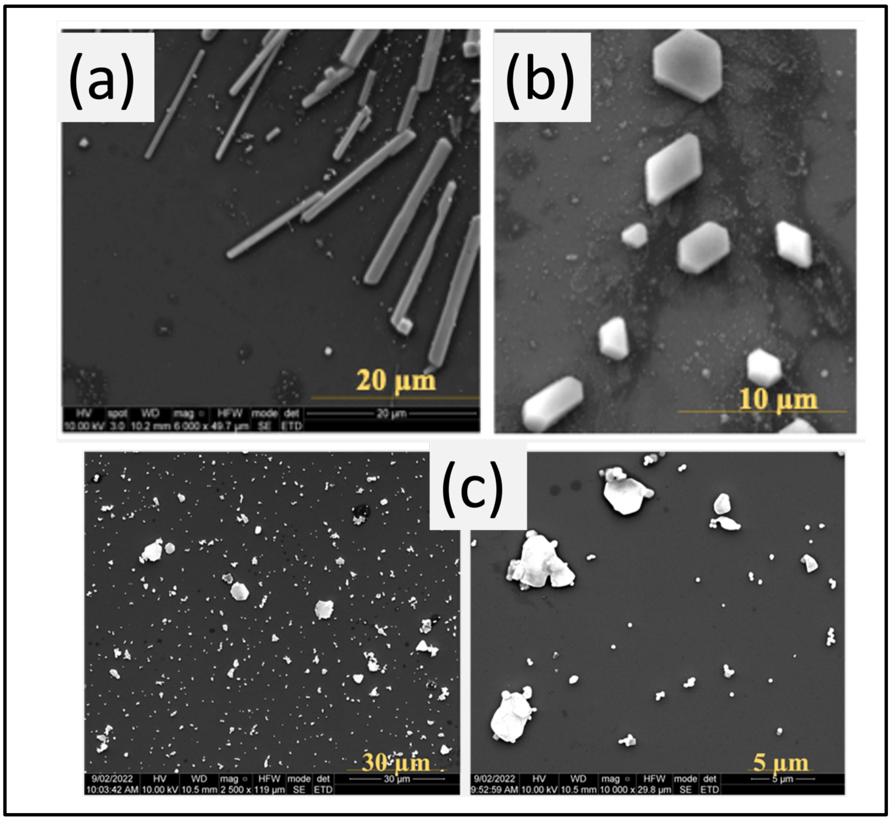

Figure S7: (a) SEM images for gold nanoparticles using a flat based tube while keeping all the parameters same as follow speed ω = 5k rpm, θ = 45˚, time = 60 mins, volume = 1 mL, concentration (c) = 3.7 mM confined mode, (λ = 254 nm) resulted in rods structure of gold. (b) SEM images for gold nanoparticles at pH of 9 while keeping all the parameters same resulted in very thick gold structure. (c) SEM images for gold processing in the VFD at θ = 0 while keeping all the parameters same as follow: for VFD processing was as follows: speed (ω) = 5k rpm, time = 60 mins, volume = 1 mL, concentration (c) = 3.7 mM confined mode, (λ = 254 nm) resulted in gold nano particles.

3.6. Changing Flow Rate - Continuous Flow

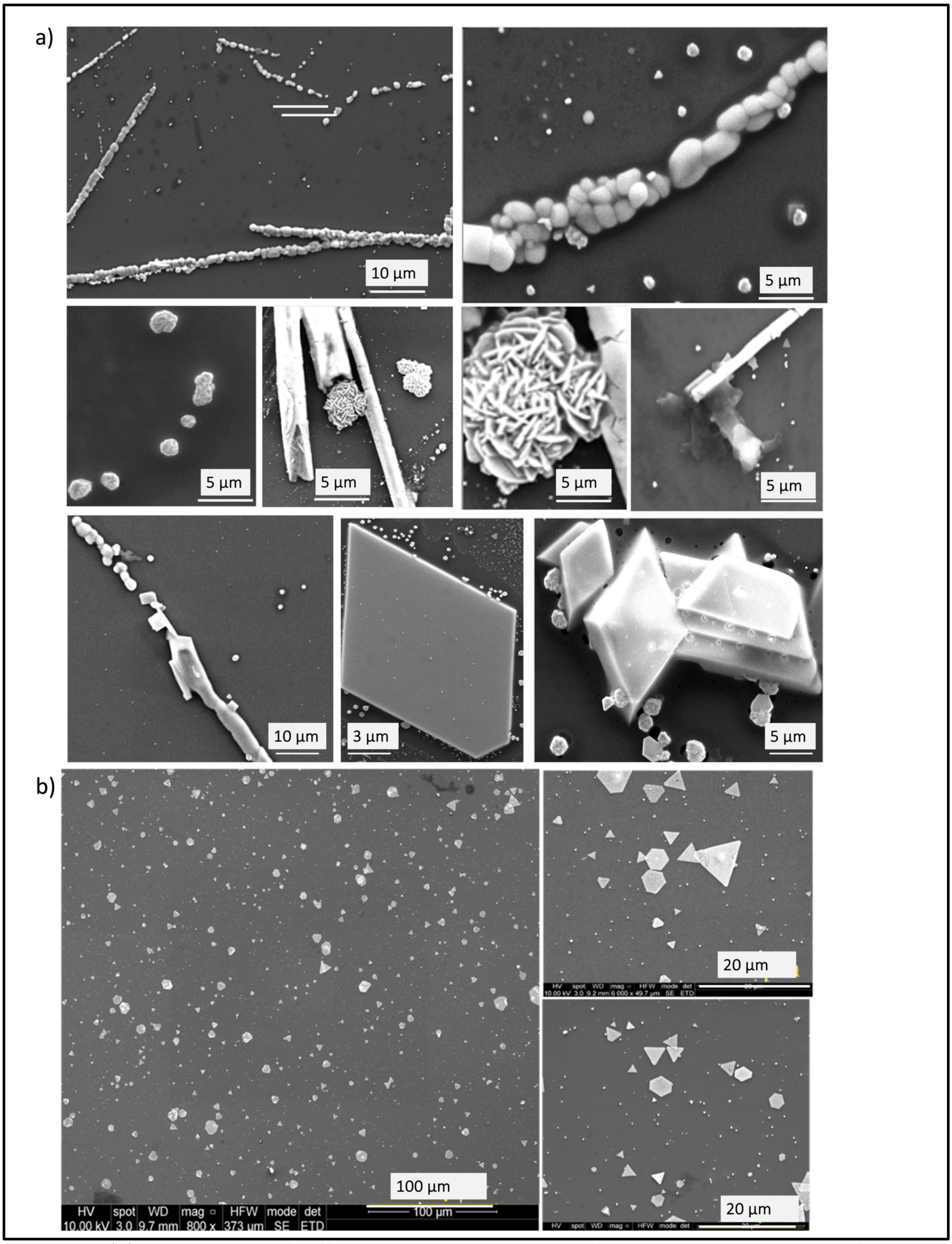

Figure S8: (a) SEM images of different gold nanoparticles formed under continuous flow with flow rate= 0.5 and 0.4 mL.min^{-1} which indicates the mechanism of nucleation and growth, b) SEM images of tringles and hexagonal of gold formed under continuous flow with flow rate rate= 0.2 and 0.3 mL.min^{-1}. The VFD processing was as follows: speed ω = 5K rpm, θ = 45˚, concentration (c) = 3.7 mM, (λ = 254 nm).

Changing Reaction Atmosphere- Under N_2 Gas Bubble

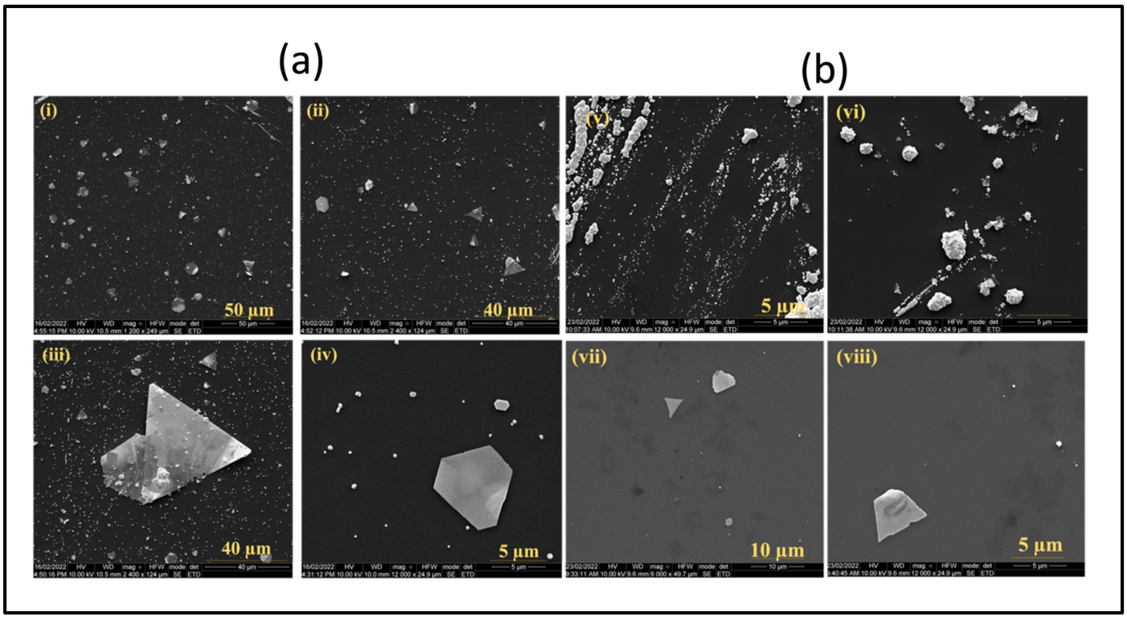

Figure S9: SEM images of (a) gold nanoparticles formed under a flow of N_2 gas in the VFD irradiated under UV (λ = 254 nm), resulting in a well-defined 2D gold structure, (b) as for (a) without UV irradiation, resulting in mainly gold nanoparticles with a few 2D gold sheets. The VFD processing was as follows: speed ω = 5K rpm, θ = 45˚, time = 60 mins, volume = 1 mL, concentration (c) = 3.7 mM confined mode.

3.7. Changing Reaction Atmosphere- Under N_2 Using a Young's Tape

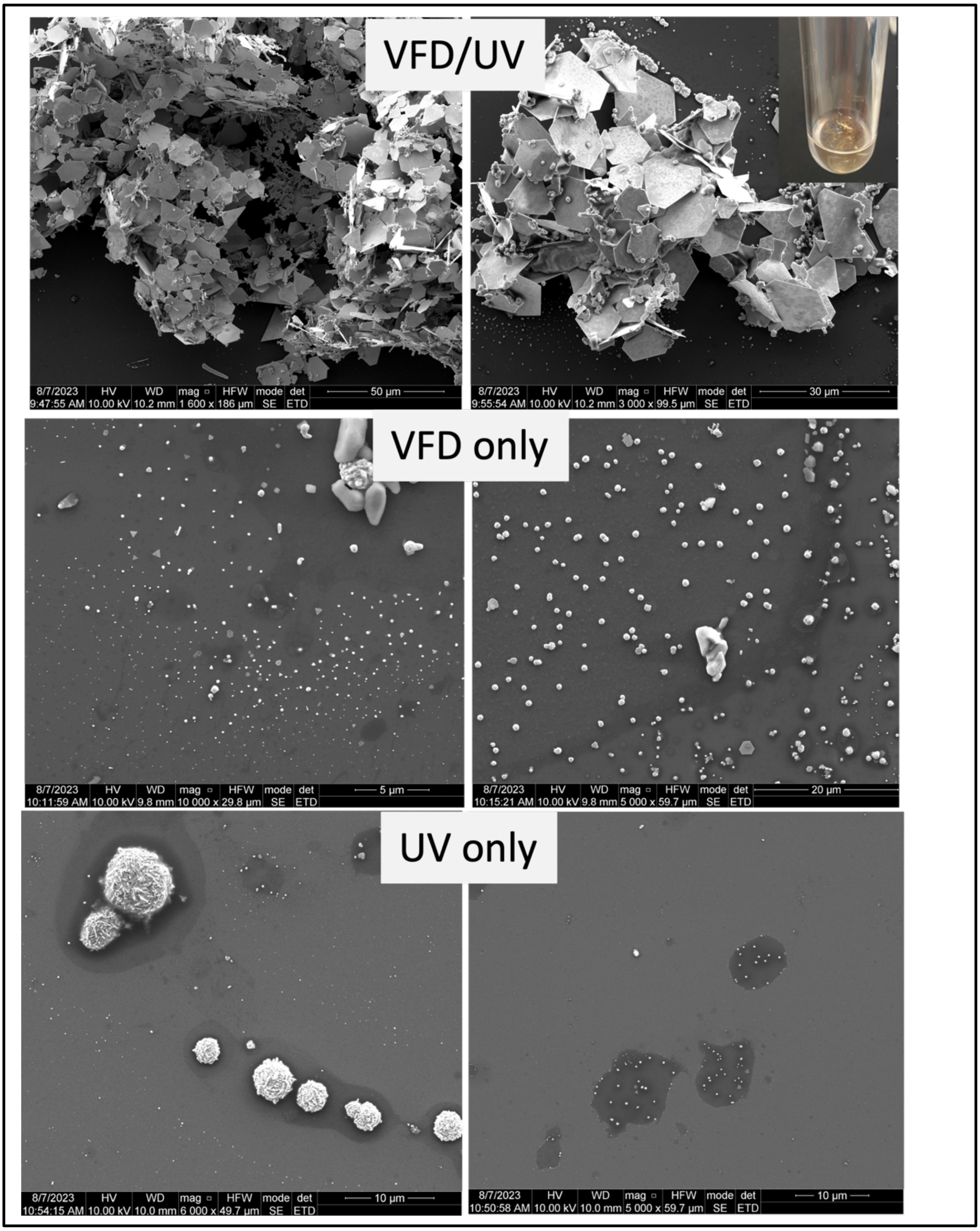

Figure S10: SEM images for gold nano particles formed under an N_2 atmosphere in a sealed Youngs tap VFD tube with UV irradiation (λ = 254 nm) affording a large amount of 2D gold sheets with some nanoparticles (top), with VFD processing without UV irradiation affording nanoparticles, and UV irradiation (no VFD) results in irregular large gold particles. The VFD processing was as follows: speed ω = 5k rpm, θ = 45˚, time = 60 mins, volume = 1 mL, concentration (c) = 3.7 mM confined mode.

3.8. VFD processing under a hydrogen atmosphere.

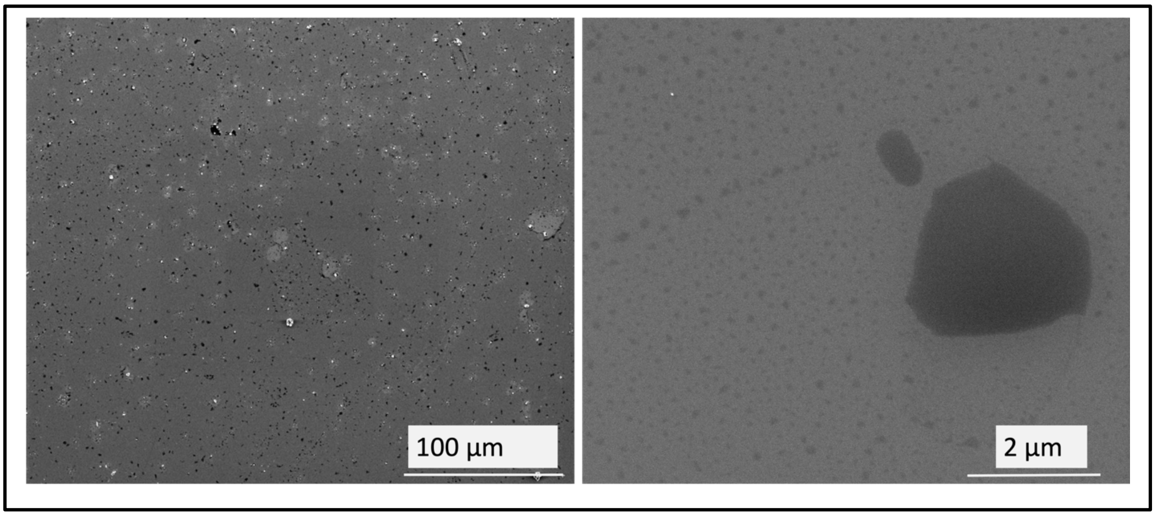

Figure S11: SEM images for gold nanoparticles formed under an H_2 atmosphere using jet feed to deliver H_2 into VFD tube. The VFD processing was as follows: speed ω = 5k rpm, θ = 45˚, time = 60 mins, volume = 1 mL, confined mode.

4. Additional Characterisations For 2D Gold Nano Sheet

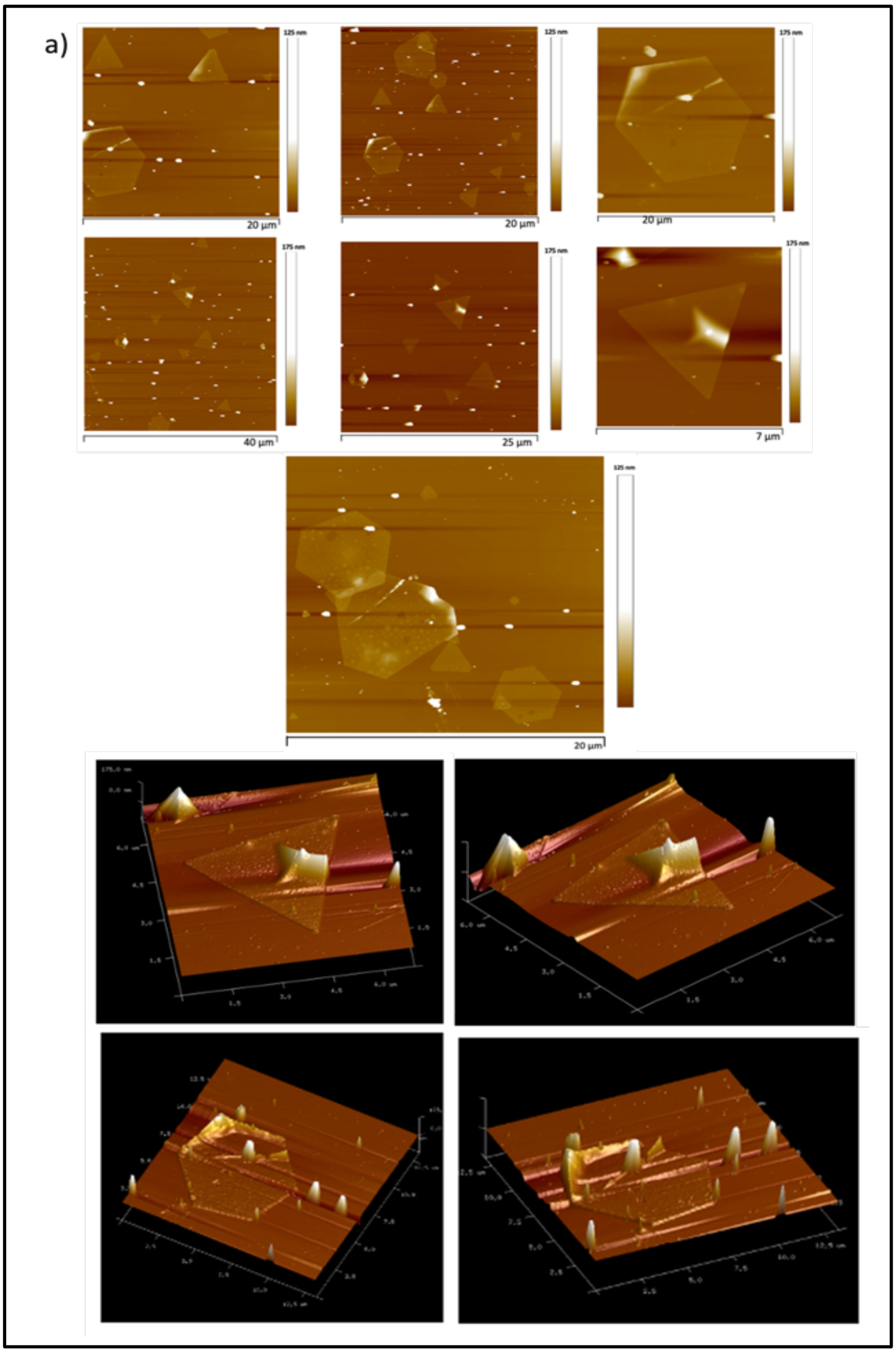

Figure S12: a) Additional AFM images for triangle and hexagonal of ultrathin gold sheets. The VFD processing was as follows: speed ω = 5K rpm, θ = 45˚, time = 60 mins, volume = 1 mL, concentration (c) = 3.7 mM, (λ = 254 nm).

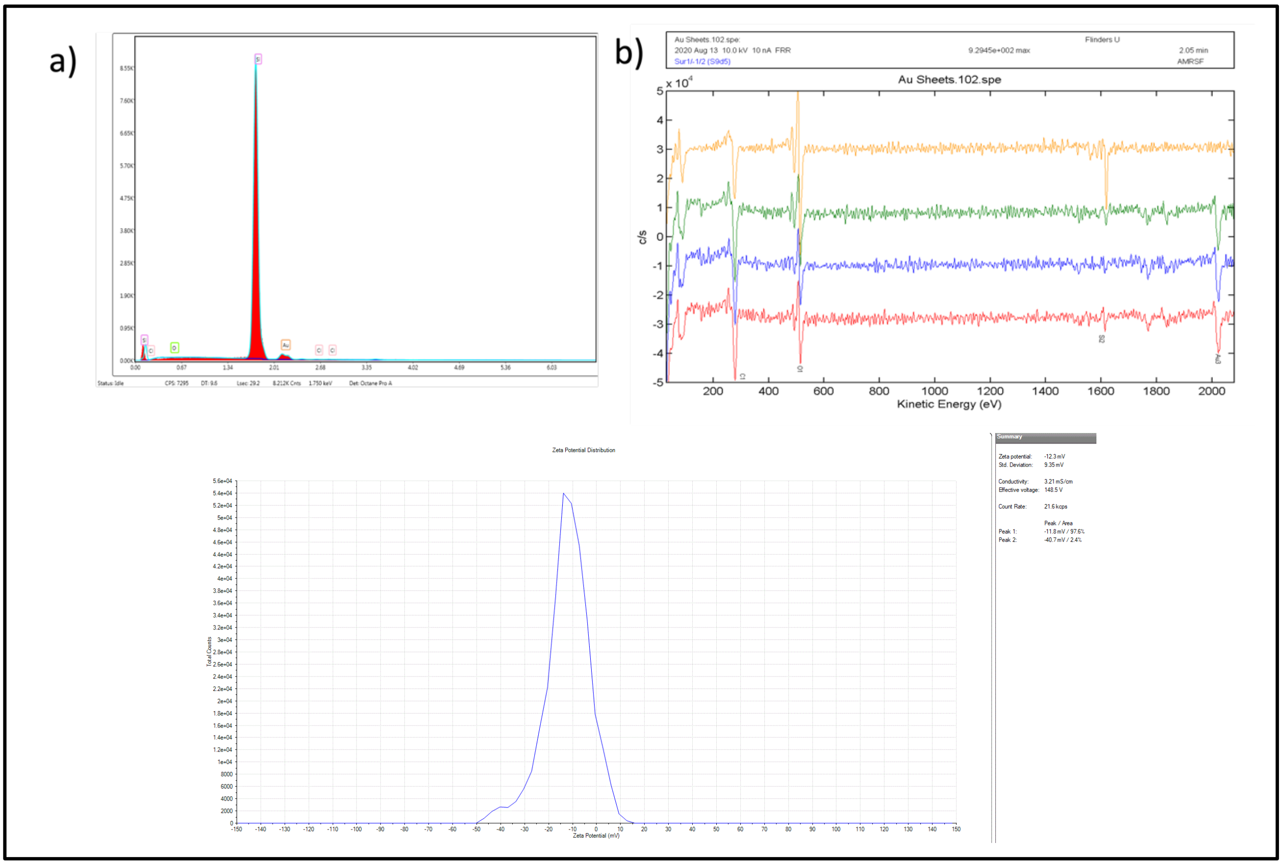

Figure S13: a) EDX spectra of the corresponding Au element being formed along with the background silicon. b) AES spectra for corresponding Au sheet at 2000 eV, with other elements such O, C and Si as a background, c) Zeta protentional value for gold nanoparticles. VFD processing was as follows: speed ω = 5k rpm, θ = 45˚, time = 60 mins, volume = 1 mL, concentration (c) = 3.7 mM, (λ = 254 nm).

5. Other Method- Sonication

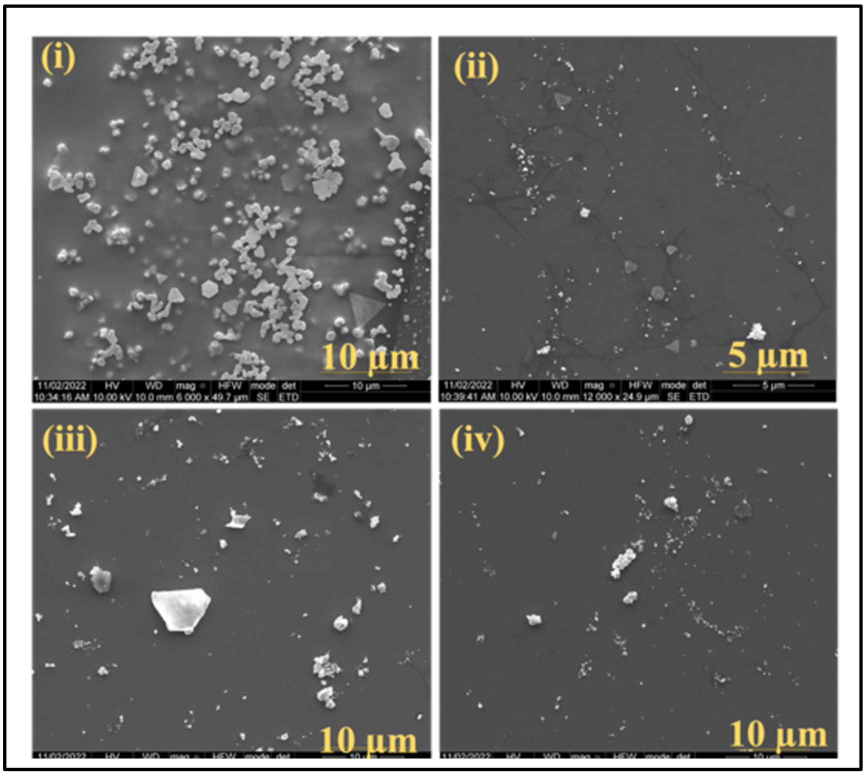

Figure S14: SEM images of gold nano particles formed under sonication with UV irradiation (i-iii), sonication without UV, (iii-v) sonication parameters as follow: time = 3 mins, volume = 1 mL, concentration= 3.7 mM, (λ = 254 nm).

6. Testing For Reactive Oxygen Species (ROS)

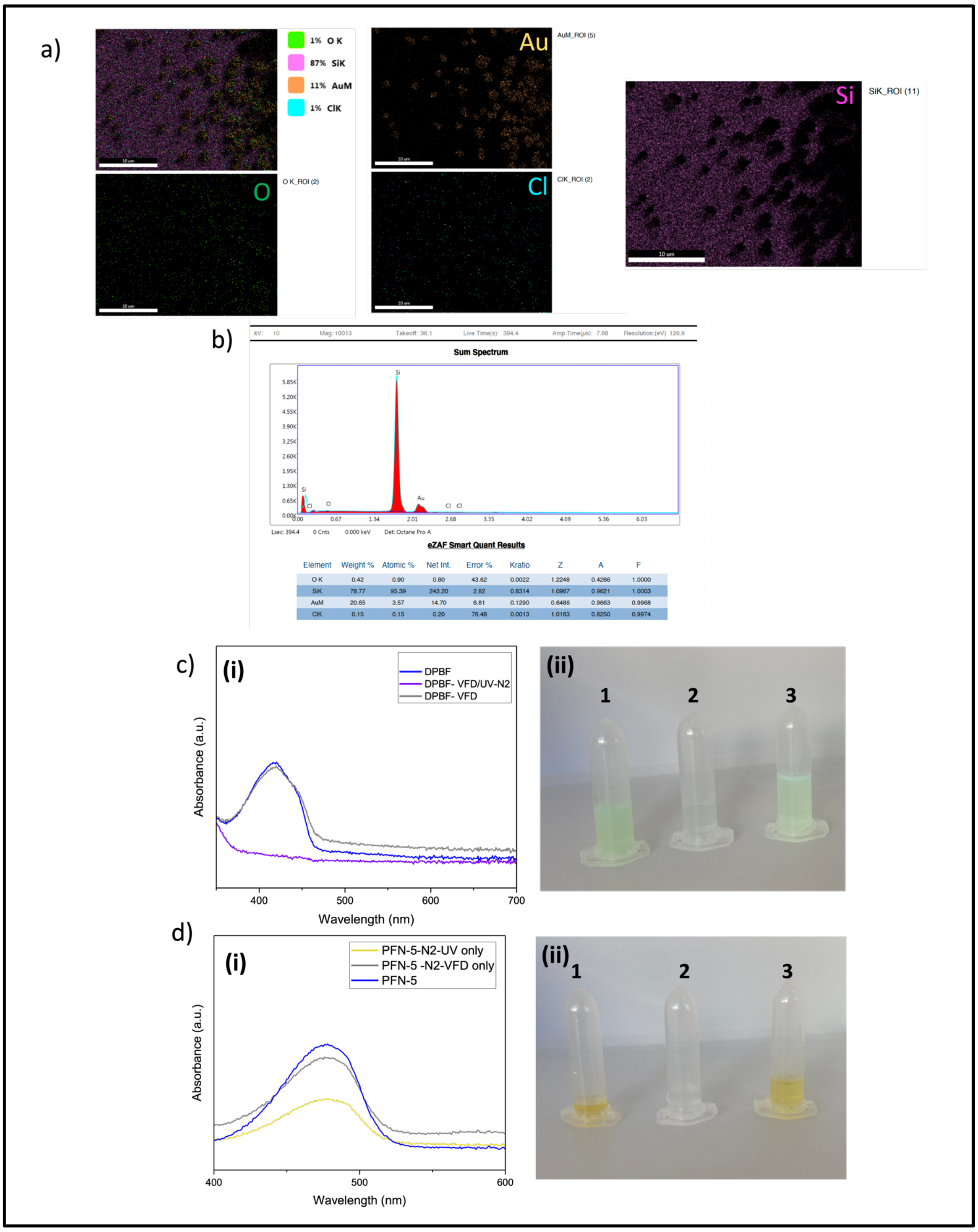

Element	Weight %	Atomic %	Net Int.	Error %	Kratio	Z	A	F
O K	0.42	0.90	0.80	43.62	0.0022	1.2248	0.4266	1.0000
SiK	78.77	95.39	243.20	2.82	0.8314	1.0967	0.9621	1.0003
AuM	20.65	3.57	14.70	6.81	0.1290	0.6486	0.9663	0.9968
ClK	0.15	0.15	0.20	76.48	0.0013	1.0163	0.8250	0.9974

Figure S15: a) EDX elemental mapping for of gold nano particles after adding auric acid at 3.7 mM to water post VFD, b) EDX spectra for gold formation after adding auric acid at 3.7 mM to water post VFD. The VFD parameters for the experiments were as follows; at speed ω = 5k rpm, θ = 45˚, time = 60 mins, volume = 1 mL, confined mode, (λ = 254 nm). c) (i) UV-VIS spectra for DPBF using the same process in air but now under nitrogen. (ii) colour of the solution 1, before VFD, 2, after VFD/UV, 3, after VFD only. d) UV-VIS spectra for PFN-5 using the same process in air but now under nitrogen. (ii) colour of the solution 1, before VFD, 2, after VFD/UV, 3, after VFD only. The VFD parameters for the experiments were as follows; speed ω = 5k rpm,

θ = 45˚, time = 60 mins, volume = 1 mL, confined mode, (λ = 254 nm), capped in confined mode processing using a Youngs VFD tape tube.

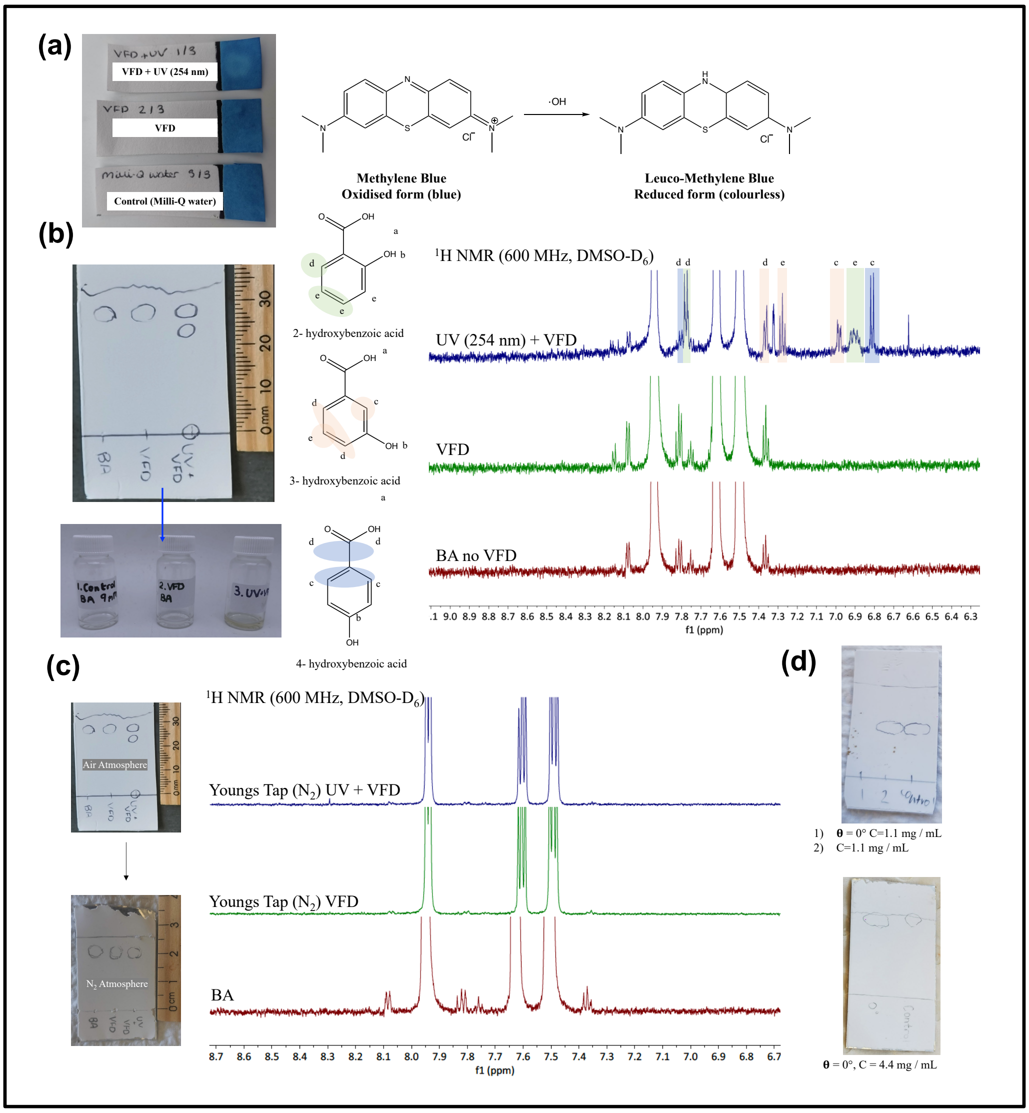

Figure S16: a) *Methylene blue test* strips of 10 microlitres of processed milli-Q water dropped onto preprepared methylene blue test strips. The VFD processing was as follows: speed ω = 7.5k rpm, angle θ = 45˚, time (t) = 20, volume (V) = 1 mL, concentration (c) = 9 mM benzoic acid, VFD processing mode = confined. b) Methylene blue reduces to form the colourless leuco-methylene blue form as in reaction outlined. b) *TLC plate* labelled as follows; Benzoic acid at concentration of 9 mM, VFD: VFD processing at with the following conditions speed ω = 7500 rpm, θ = 45˚ time (t) = 20, volume (V) = 1 mL, concentration, c) = 9 mM BA Mode = Confined, UV + VFD: parameters consistent as in 'VFD' with UV (λ = 254 nm) lamps. b) Labelled borosilicate glass bottles containing samples, noting the yellow colour of the sample where UV has been used in conjunction with VFD processing (7.5 k rpm). The bottle labels are as follows; 1. BA 2. VFD 3. UV + VFD and are consistent with the reaction conditions outlined in a). c) ^{1}H NMR spectra of the samples; BA, VFD and UV + VFD, under air, analysed in DMSO-D6 using a 600 MHz NMR spectrometer. The labelled peaks are as follows: 2-hydroxybenzoic acid in

green, 3-hydroxybenzoic acid in orange and 4-hydroxybenzoic acid in blue. c) TLC plate labelled as Air and Nitrogen atmosphere with all following conditions consistent between the two as a direct comparison between the air; BA: Benzoic acid at concentration of 9 mM, VFD: VFD processing at with the following conditions speed ω = 7.5k rpm, θ = 45˚ time (t) = 20, volume (V) = 1 mL, concentration (c) = 9 mM BA Mode = Confined, UV + VFD: parameters consistent as in 'VFD' with UV (254 nm) lamps. b) 1H NMR spectra of the samples; BA, VFD and UV + VFD, under nitrogen atmosphere as prepared using a young's tap tube, analysed in DMSO-D6 using a 600 MHz NMR spectrometer d) TLC plate labelled as 1), θ = 0˚ and 2), θ = 45˚ with all following conditions consistent Benzoic acid at concentration of 9 mM, VFD: VFD processing at with the following conditions speed ω = 7.5k rpm, angle (t) = 20, volume (V) = 1 mL, concentration (c) = 9 mM. b) All parameters as in a) with θ = 0˚and the concentration of 36 mM.

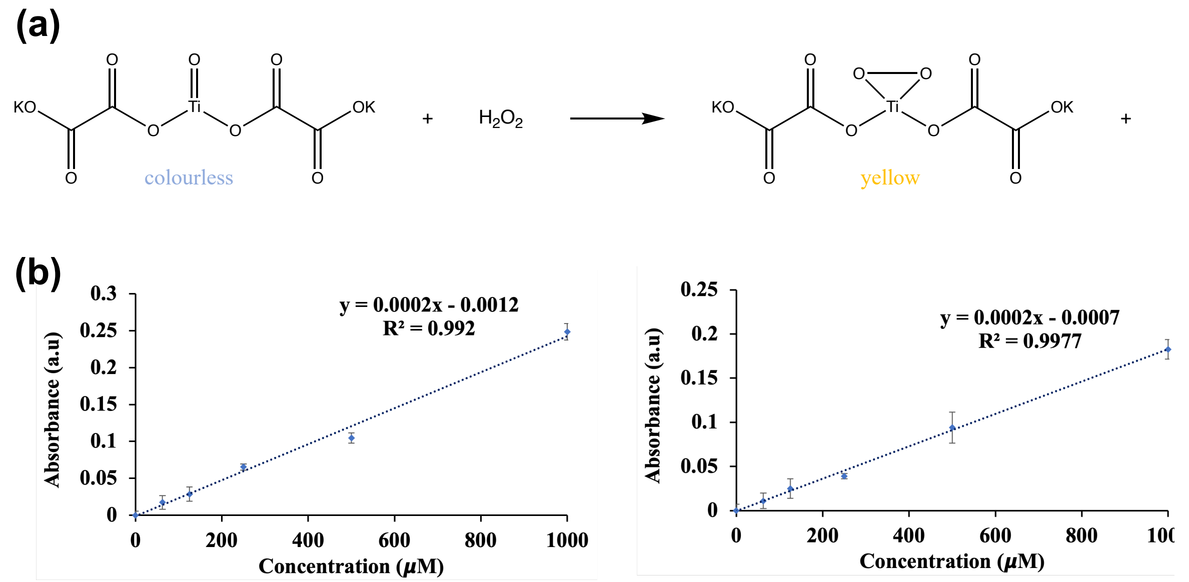

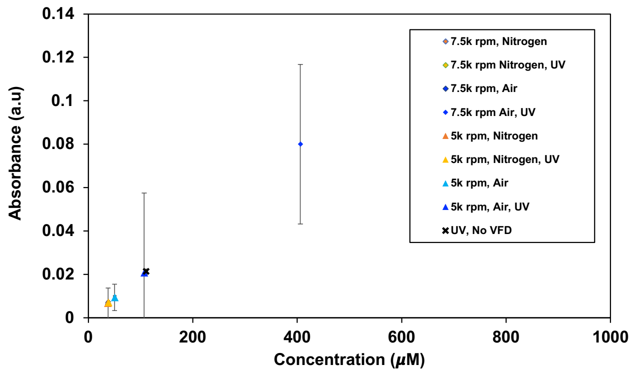

Conditions	Concentration (μM)
7.5k rpm, Nitrogen	-132.3±24.6
7.5k rpm, Nitrogen, UV	-25.6±83.1
7.5k rpm, Air	37.6±36.9
7.5k rpm, Air, UV	406.0±183.8
5k rpm, Nitrogen	36.8±45.1
5k rpm, Nitrogen, UV	38.5±11.6
5k rpm, Air	50.2±30.4
5k rpm, Air, UV	106.8±28.4
UV, No VFD	110.2±30.4

Figure S17: Quantification of hydrogen peroxide (H_2O_2) was completed though the Potassium Titanium Oxalate (PTO) method as outlined in equation (a). (b) Hydrogen peroxide potassium titanium oxalate assay prepared on day of processing for a) 7.5 rpm and b) 5k rpm. (c) Concentration of hydrogen peroxide, as per potassium titanium oxalate analysis method, outlining parameters tested. All reactions are kept consistent as ω = 7.5k rpm/5k rpm, angle θ = 45˚ time = 5, volume = 2 mL. The atmosphere was altered to be nitrogen (N_2) or air as prepared in a young's tap tube using a Schlenk line, and UV (λ = 254 nm) irradiation was used. The UV no VFD experiment was prepared using the tube at an angle of 0° relative to the horizon.

7. Hydrogen Detection

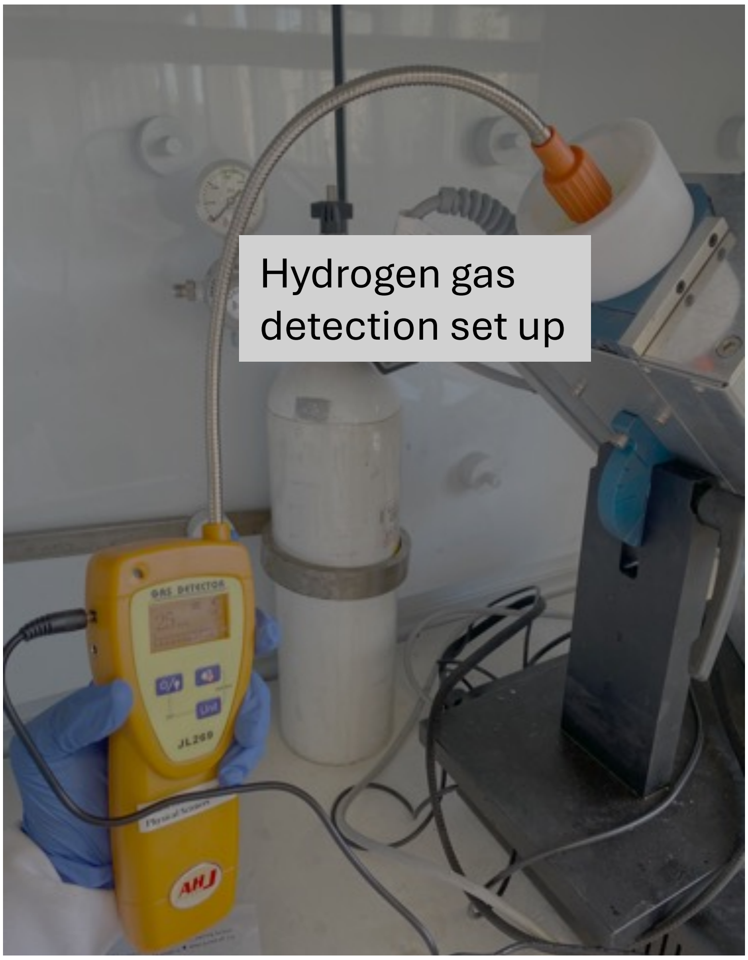

condition	H2o/air/UV	H2o/air/no UV	H2o/N_2/UV	H2o/N_2/no UV	Au/air/UV	Au/air/no UV	Au/N_2/UV	Au/N_2/no UV
Concentration of H_2	200 ppm	200 ppm	300 ppm	200 ppm	500 ppm	400 ppm	2000 ppm	2000 ppm

Figure S18: A Digital Gas Sniffer, Model: JL269, flammable gas detector with a flexible goose neck probe was used to detect hydrogen gas. The probe close to the open end of the VFD tube. The concentration of hydrogen shows in ppm after 5 min VFD processing. The table shows a concentration of hydrogen gas in ppm under different condition. VFD parameters as follow: speed ω = 5k rpm, θ= 45˚, time = 5 mins, volume = 1 mL of MilliQ-water or concentration of auric acid (c) = 3.7 mM confined mode, (λ = 254 nm) under air or nitrogen atmosphere.

8. Enhancement of CE in the VFD

Coated The Tube with a Layer of Silica Xerogel in the formation of nano gold

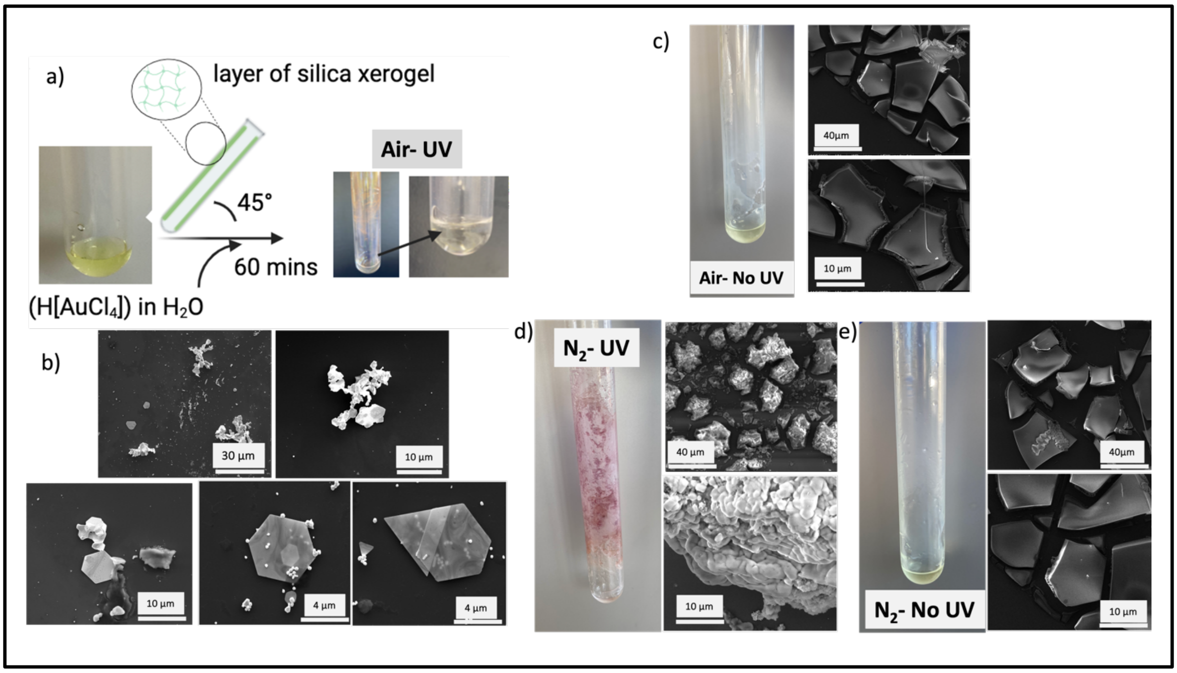

Figure S19: Enhancment of CE in the VFD was tested by, (a) Coated the tube with a layer of silica xerogel setup processing and colour of the resulted solution after processing in the VFD in air under UV irradiation (b) SEM images for the resulting formation of 2D gold in air VFD under UV irradiation, (c) same reaction process in the VFD in air in the absent of UV irradiation, (d) processing under nitrogen with UV irradiation, (e) reaction under nitrogen in the absent of UV. VFD parameters as follow: speed ω = 5k rpm, θ = 45˚, time = 60 mins, volume = 1 mL, confined mode, (λ = 254 nm)

9. Author Contributions

B.A. performed all the VFD experiments, data analysis for SEM, EDX, XRD, Auger and XPS, UV-Visible spectroscopy, and DPBF, PFN-5 and xerogel studies, and H_2 detection. Z.G. carried out the BA experiments, hydroxyl radical and hydrogen peroxide detection. X.C. and T.M. carried out the TEM analysis. K.V. carried out the AFM analysis. B. A and C.L.R. developed the overall CE mechanism. C.L.R. coordinated the research and developed the model for the fluid behaviour. The primary content of the manuscript was written by B.A., and Z.G. wrote the BA and H_2O_2 contents following the completing of the first full draft by B.A. and C.L.R. All authors have given approval to the final version of the manuscript.

.
